# CDK6 Degradation Is Counteracted by p16^INK4A^ and p18^INK4C^ in AML

**DOI:** 10.3390/cancers14061554

**Published:** 2022-03-18

**Authors:** Belinda S. Schmalzbauer, Teresemary Thondanpallil, Gerwin Heller, Alessia Schirripa, Clio-Melina Sperl, Isabella M. Mayer, Vanessa M. Knab, Sofie Nebenfuehr, Markus Zojer, André C. Mueller, Frédéric Fontaine, Thorsten Klampfl, Veronika Sexl, Karoline Kollmann

**Affiliations:** 1 Institute of Pharmacology and Toxicology, University of Veterinary Medicine Vienna, 1210 Vienna, Austria; belinda.schmalzbauer@vetmeduni.ac.at (B.S.S.); teresemary.thondanpallil@merck.com (T.T.); alessia.schirripa@vetmeduni.ac.at (A.S.); clio.sperl@hotmail.com (C.-M.S.); isabella.mayer@vetmeduni.ac.at (I.M.M.); vanessa-maria.knab@vetmeduni.ac.at (V.M.K.); sofie.nebenfuehr@vetmeduni.ac.at (S.N.); markus.zojer@vetmeduni.ac.at (M.Z.); thorsten.klampfl@vetmeduni.ac.at (T.K.); veronika.sexl@vetmeduni.ac.at (V.S.); 2 Division of Oncology, Department of Medicine I, Medical University of Vienna, 1090 Vienna, Austria; gerwin.heller@meduniwien.ac.at; 3 CeMM—Research Center for Molecular Medicine of the Austrian Academy of Sciences, 1090 Vienna, Austria; andre.mueller@thermofisher.com (A.C.M.); ffontaine@cemm.oeaw.ac.at (F.F.)

**Keywords:** CDK6, AML, degrader, INK4, p16, p18, *Cdkn2a*, *Cdkn2c*

## Abstract

**Simple Summary:**

CDK4/6 kinase inhibitors show promising results in various subtypes of AML, which has been primarily assigned to the inhibition of CDK6. To bypass therapeutic resistances and tackle the kinase-dependent, as well as kinase-independent, functions of CDK6, new CDK6 degraders have been developed. Here, we present insights into the mechanistic requirements for the efficacy of a CDK6-specific degrader in AML. We show that the presence and levels of the INK4 proteins p16^INK4A^ and p18^INK4C^ determine the extent of CDK6 degradation. Our study reveals the importance of INK4 protein levels as predictive markers for CDK6-targeted therapy in AML.

**Abstract:**

Cyclin-dependent kinase 6 (CDK6) represents a novel therapeutic target for the treatment of certain subtypes of acute myeloid leukaemia (AML). CDK4/6 kinase inhibitors have been widely studied in many cancer types and their effects may be limited by primary and secondary resistance mechanisms. CDK4/6 degraders, which eliminate kinase-dependent and kinase-independent effects, have been suggested as an alternative therapeutic option. We show that the efficacy of the CDK6-specific protein degrader BSJ-03-123 varies among AML subtypes and depends on the low expression of the INK4 proteins p16^INK4A^ and p18^INK4C^. INK4 protein levels are significantly elevated in KMT2A-MLLT3+ cells compared to RUNX1-RUNX1T1+ cells, contributing to the different CDK6 degradation efficacy. We demonstrate that CDK6 complexes containing p16^INK4A^ or p18^INK4C^ are protected from BSJ-mediated degradation and that INK4 levels define the proliferative response to CDK6 degradation. These findings define INK4 proteins as predictive markers for CDK6 degradation-targeted therapies in AML.

## 1. Introduction

Acute myeloid leukaemia (AML) is an aggressive disease that comprises a highly heterogeneous group of genetically distinct subtypes. The initiation and propagation of AML is driven by leukemic stem cells (LSCs), which are able to self-renew and give rise to malignant myeloid cells [1]. A genome-scale CRISPR/Cas9 screen of 342 cancer cell lines revealed that AML cells are highly dependent on cyclin-dependent kinase 6 (CDK6) but not on its close homologue cyclin-dependent kinase 4 (CDK4) [2,3]. Further support for the key role of CDK6 in AML stems from the finding that CDK6 is a prognostic biomarker for AML [4]. The chromosomal translocations of AML that have been linked to CDK6 addiction include t(8;21) generating RUNX1-RUNX1T1 (previously AML1-ETO), t(9;11) inducing KMT2A-MLLT3 (previously MLL-AF9) and t(5;11) leading to FLT3-ITD [5,6,7,8,9]. In addition, CDK6 has been implicated as a key regulator of LSC functions [10]. As such, CDK6 regulates the transcription of early growth response protein 1 (EGR1) and interacts with runt-related transcription factor 1 (RUNX1) to block RUNX1-dependent transcriptional effects. The involvement of CDK6 in these processes underscores its potential as a target for AML therapy [5,10,11].

The ATP-competitive small molecule compound palbociclib inhibits the enzymatic functions of CDK6 and CDK4 and has been FDA approved for the treatment of breast cancer patients. However, insensitivity to palbociclib commonly occurs [12]. Primary resistance to palbociclib has been associated with high levels of p16^INK4A^ [13,14]. P16^INK4A^ is an intrinsic inhibitor of CDK4 and CDK6 and together with p15^INK4B^, p18^INK4C^ and p19^INK4D^, it belongs to the INK4 protein family [15,16]. The binding of INK4 proteins to CDK4 or CDK6 induces conformational changes to the cell cycle kinases reducing D-type cyclin binding, which decelerates G1 to S phase cell cycle transition. In breast cancer cells, a high p16^INK4A^ level confers drug resistance but low levels are not consistently associated with sensitivity to palbociclib [13,14]. In AML, INK4 proteins contribute to disease progression and the downregulation of p16^INK4A^ correlates with worse survival prognosis for AML patients [17,18].

Considering that CDK6 not only functions as a cell cycle kinase but also regulates transcriptional processes in a kinase-dependent and kinase-independent manner, it was of great interest to establish a strategy that targets all of the functions of CDK6 [19]. Homolog selective pharmacological CDK6 degraders that exploit the cell intrinsic proteasomal machinery to degrade the CDK6 protein have been developed. Initial studies showed that these compounds successfully degrade CDK6 in leukemic cells in vitro and *in vivo*, which results in a clear advantage over CDK4/6 kinase inhibitors [3,20]. A single KMT2A-AFF1+ cell line has been employed in the in vitro study, which restricts conclusions in a broader context [3].

Here, we extended these findings and studied the effects of CDK6 degrader efficacy in two CDK6-dependent AML subtypes harbouring either KMT2A-MLLT3 or RUNX1-RUNX1T1. We took advantage of a novel haematopoietic progenitor cell model (HPC^LSK^s) to study CDK6 degrader efficacy in a genetically defined setting of AML subtypes. One major advantage of this cell line model is its leukemic stem/progenitor state. The involvement of p16^INK4A^ in palbociclib resistance and the role of INK4 proteins in AML disease progression require a systematic investigation of INK4 proteins within the context of CDK6 degradation in distinct AML subtypes. We identified p16^INK4A^ and p18^INK4C^ as dominant CDK6 binding partners that counteract pharmacological protein degradation.

## 2. Materials and Methods

### 2.1. RNA Expression Data from Human AML Patients

Affymetrix HG-U133_plus_2.0 data were obtained from the ArrayExpress database (https://www.ebi.ac.uk/arrayexpress/experiments/E-MTAB-3444/, last accessed 10 March 2022) and GEO database (GSE13159). Raw microarray data were processed, normalised and log2 transformed using the frozen robust multiarray (fRMA, version 1.42.0) algorithm and R 4.0.4 software. Log2 transformed data were batch corrected using the ComBat function of the R package SVA (version 3.38.0), followed by the calculation of differential expressions using the lmFit function of the limma package (version 3.46.0). An FDR < 0.05 was used as the cut-off for statistical significance.

### 2.2. Cell Line Establishment and Cell Culture

Haematopoietic progenitor cells were generated from mouse bone marrow with lineage-negative and lineage-positive selection for c-Kit and Sca-1 (HPC^LSK^) according to a recently established protocol [21]. HPC^LSK^ cells were then retrovirally transformed with either pMSCV-KMT2A-MLLT3 IRES (Venus) or pMSCV-RUNX1-RUNX1T19a-IRES (GFP) vectors using Platinum-E Retroviral Packaging Cells and TurboFect (Thermo Fisher Scientific, Waltham, MA, USA) according to the manufacturer’s instructions. HPC^LSK^ AML cells were selected for GFP or Venus expression using fluorescence activated cell sorting analysis (FACS) with the BD FACS Aria™ III (BD Biosciences, Franklin Lakes, NJ, USA). The cell culture conditions of the HPC^LSK^ AML cell lines were maintained according to the published protocol [21]. Shortly afterwards, the cells were kept in the Iscove’s Modified Dulbecco Medium (IMDM; Sigma-Aldrich, St. Louis, MO, USA), which was supplemented with 5% foetal bovine serum (Gibco, Thermo Fisher Scientific, Waltham, MA, USA), 1.5 × 10^−4^ M 1-thiolglycerol, 1% penicillin/streptomycin (Sigma), 2 mM L-glutamine (Sigma-Aldrich, St. Louis, MO, USA), 2% SCF (in-house preparation) and 12.5 ng/mL interleukin-6 (IL-6; a gift from the Research Institute of Molecular Pathology (IMP), Vienna). The HPC^LSK^ AML cells were cultured on 1% agarose (peqGOLD Universal Agarose, VWR, Darmstadt, Germany) coated culture plates in a 5% CO_2_ humidified incubator. The BCR-ABL^p185+^ cells were cultivated as described previously [22]. Human AML cell lines NOMO-1 (RRID:CVCL_1609), THP-1 (RRID:CVCL_0006), MOLM13 (RRID:CVCL_2119) and MV4-11 (RRID:CVCL_0064) were cultured in an RPMI-1640 growth medium (Sigma-Aldrich, St. Louis, MO, USA) that was supplemented with 10% foetal bovine serum (Gibco, Thermo Fisher Scientific, Waltham, MA, USA) and 1% penicillin/streptomycin (Sigma-Aldrich, St. Louis, MO, USA).

### 2.3. RNA Isolation and RT-qPCR

RNA was extracted using the RNeasy Mini Kit (Qiagen, Venlo, Netherlands) or the RNA-Solv^®^ Reagent (Omega Bio-tek, Norcross, GA, USA) according to the manufacturer’s instructions. For reverse transcription, the iScript cDNA Synthesis Kit was used (Bio-Rad, Hercules, CA, USA). Real-time quantitative PCRs (RT-qPCR) were performed with the SsoAdvanced Universal SYBR Green Supermix (Bio-Rad, Hercules, CA, USA). The QPCRs were performed on a CFX96 real time PCR machine (Bio-Rad, Hercules, CA, USA). The QPCR primer sequences are summarised in Table S1.

### 2.4. RNA Sequencing

The RNA sequencing dataset from a different, still unpublished, study with a separate focus was used to look specifically at the expression of the INK4 genes. RNA was isolated from the HPC^LSK^ WT cells and AML cells that were harbouring KMT2A-MLLT3 or RUNX1-RUNX1T1. Sequencing libraries were prepared using the NEB Next Ultra II kit. Sequencing was performed on an Illumina NovaSeq system (Illumina, San Diego, CA, USA) in a 50-bp paired-end fashion. Demultiplexed reads were aligned against the GRCm38 primary assembly mouse reference genome using STAR 2.7.6a [23]. The gene model used was the Gencode M25 primary assembly annotation [24]. Reads aligned across genes were counted using the featureCounts function from the subred 2.0.1 package [25]. Differential gene expression analysis was performed in R (Version 4.0.3) with the DESeq2 package [26,27]. Normalised fragment counts were calculated by multiplying raw fragment counts with size factors, as implemented in DESeq2.

### 2.5. Immunoblot Analysis

Proteins were isolated using an RIPA buffer composed of 150 mM NaCL, 50 mM Tris HCl pH 8 and 0.1% SDS, which was supplemented with a proteinase inhibitor cocktail (cOmplete™, Roche, Basel, Switzerland), and shaking for 30 min and 20 min centrifugation at 13,000 rpm at 4 °C. The concentrations of the whole-cell lysates were measured colourimetrically (Pierce™ BCA Protein Assay Kit, Thermo Fisher Scientific) on an EnSpire^®^ Multimode Plate Reader (Perkin Elmer, Waltham, MA, USA). Protein lysates were incubated at 95 °C with a Laemmli buffer before being loaded onto the 10% or 12% sodium dodecyl sulphate polyacrylamide (SDS-PAGE) gel. Separated proteins were transferred to menthol activated Immobilon^®^-P polyvinylidene difluoride membranes (PVDF; Merck, Darmstadt, Germany) using overnight blotting with 0.2 mA for 18 h followed by 0.4 mA for 5 h at 4 °C. The membranes were blocked with 5% BSA in a Py-TBST buffer and incubated overnight with the primary antibodies in 3% BSA. Secondary antibodies were incubated for 1 h at room temperature (RT). The chemiluminescence of the membranes was measured with the ChemiDoc™ Imaging System (Bio-Rad, Hercules, CA, USA) after incubation with 20× LumiGLO^®^ Reagent and 20× Peroxide (Cell Signaling Technology, Danvers, MA, USA). The densitometry quantification of the signals was performed with the Image Lab 5.2.1. software (Bio-Rad, Berkeley, CA, USA). Table S2 summarises the antibodies that were used.

### 2.6. CDK6 Degrader and CDK4/6 Inhibitor Treatments

BSJ-03-123 (BSJ) was purchased from MedChemExpress LLC (Princeton, NJ, USA) [3]. BSJ was used at concentrations ranging from 0.75 to 9 µM, as indicated in the respective figure. Cells were seeded for the treatments at a density of 0.5 × 10^6^ cells per mL and analysed at different time-points, as indicated in the respective figures. The BSJ media was renewed every day. Palbociclib (PD-0332991) was obtained from Pfizer (New York City, NY, USA).

### 2.7. Flow Cytometry

Flow cytometry analyses were performed on CytoFLEX S (Beckman Coulter, Inc., Brea, CA, USA). The oncogene expression of KMT2A-MLLT3 (Venus) and RUNX1-RUNX1T1 (GFP) was detected in the FITC channel. Cell cycle analysis was performed using propidium iodide (PI) through the incubation of the cell suspension in a PI buffer that was supplemented with a 0.1% Na citrate dehydrate, 0.1% Triton-X-100 (Sigma-Aldrich, St. Louis, MO, USA), 0.1% RNAse (Sigma-Aldrich, St. Louis, MO, USA) and 20 µg/mL PI (Sigma-Aldrich, St. Louis, MO, USA) by incubating for 30 min at 37 °C followed by immediate FACS analysis in the PE channel. Cell surface staining was performed to measure myeloid differentiation using an antibody against the surface marker Gr-1 (Ly-6G) (listed in the supplementary methods section). Cells were washed with PBS and incubated with the antibody for 1 h at 4 °C. Cells were then again washed with PBS and resuspended in PBS for the flow cytometry analyses.

### 2.8. Immunoprecipitation (IP) and Mass Spectrometry (MS) Analysis

Cell lysis was performed using an egg lysis buffer composed of 0.1% NP-40, 50 mM N-2-hydroxyethylpiperazine-N′-2-ethanesulfonic acid, 250 mM NaCl and 5 mM EDTA in the presence of 0.4 mM Na_3_VO_4_, 1 mM phenylmethylsulfonyl fluoride (PMSF), 2 mM NaF and a protease inhibitor cocktail (Roche, Basel, Switzerland). Protein concentration was measured colourimetrically (Pierce™ BCA Protein Assay Kit, Thermo Fisher Scientific, Waltham, MA, USA) on an EnSpire^®^ Multimode Plate Reader (Perkin Elmer, Waltham, MA, USA). Then, 600 µg of protein and 16 µg of the CDK6 C-21 antibody were incubated overnight at 4 °C under rotation. Protein–antibody complexes were then incubated with 40 µL Pierce Protein A/G Agarose Beads (Thermo Fisher Scientific, Waltham, MA, USA) for 2 h at 4 °C under rotation. For normal Ips, 30 µL of 4× Laemmli buffer was added to the beads for elution at 95 °C for 10 min. For IP-MS analysis, the agarose beads were washed in an IP buffer containing 50 mM HEPES pH 8.0, 100 mM KCl, 2 mM EDTA, 10% glycerol and 0.1% NP-40 substitute. For the elution of the protein complexes, the beads were incubated in 50 µL of a 2% SDS elution buffer that was supplemented with 50 mM HEPES pH 8.0, 150 mM NaCl and 5 mM EDTA at 95 °C for 10 min. An equivalent of 30 µg of total protein input, supernatant (SN) and 10% of the IP eluate were separated and analysed using 10% SDS-PAGE gel, as described in the Immunoblot Analysis. In total, 90% of the IP eluate was submitted for analysis based on liquid chromatography coupled with tandem mass spectrometry (LC-MS/MS) proteomics, as described in the supplementary methods section. The IP-MS experiment was performed at the Proteomics Facility of the Research Centre for Molecular Medicine of the Austrian Academy of Sciences (CeMM, Vienna, Austria).

### 2.9. Statistical Analysis, Data Visualisation and Graphical Design

The data illustration and statistical analysis were performed using GraphPad Prism 8.4.3 software (GraphPad, San Diego, CA, USA http://www.graphpad.com, last accessed 12 March 2022). When not indicated differently in the figure legends, the data are presented as mean values + SEM. The *p*-values from the Student’s *t*-test are presented as follows: * *p* < 0.05; ** *p* < 0.01; and *** *p* < 0.001. The false discovery rates (FDR) from the RNA expressions of the human AML patients are presented as follows: * FDR < 0.05; ** FDR < 0.01; and *** FDR < 0.001. The experimental outlines and mechanistic models were created using BioRender.com.

## 3. Results

### 3.1. Differential INK4 Expression in KMT2A-MLLT3+ and RUNX1-RUNX1T1+ AML

**Figure 1:**
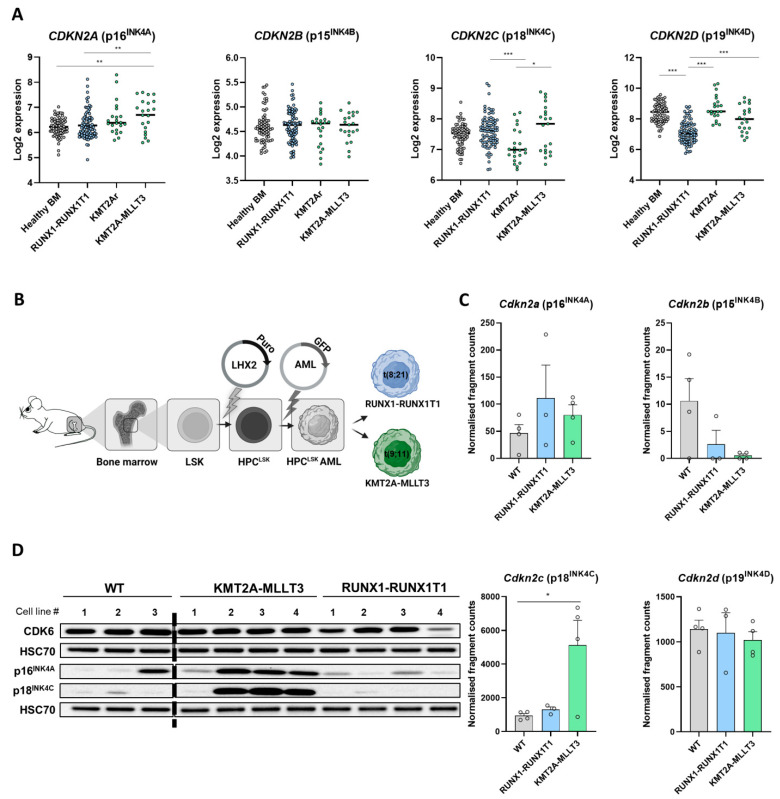
INK4 protein levels differ among AML subtypes. (**A**) The RNA expressions of INK4 genes from human AML patient microarray datasets were analysed: healthy bone marrow, BM (*n* = 74); RUNX1-RUNX1T1, t(8;21) (*n* = 86); KMT2A rearrangements, KMT2Ar (11q23) (*n* = 22); KMT2A-MLLT3, t(9;11) (*n* = 21). The KMT2Ar contained no KMT2A-MLLT3 samples. The line represents the median; * FDR < 0.05; ** FDR < 0.01; *** FDR < 0.001. (**B**) A schematic representation of experimental procedure. Murine bone marrow cells were isolated and immortalised with an LHX2 plasmid vector for generating HPC^LSK^ lines. HPC^LSK^ cells were then transformed by the integration of human RUNX1-RUNX1T1 t(8;21) (GFP) or KMT2A-MLLT3 t(9;11) (Venus) plasmid vectors. (**C**) The RNA expression of INK4 genes in the HPC^LSK^ WT (*n* = 4), RUNX1-RUNX1T1+ (*n* = 3) and KMT2A-MLLT3+ (*n* = 4) cells taken from a RNA sequencing dataset: * *p* = 0.0299. (**D**) The immunoblot of the different biological replicates of HPC^LSK^ WT (*n* = 3), KMT2A-MLLT3+ (*n* = 4) and RUNX1-RUNX1T1+ (*n* = 4) cells detecting CDK6, p16^INK4A^ and p18^INK4C^. HSC70 served as a loading control. The uncropped immunoblots are depicted in Figure S5 and the densitometry quantification is presented in Figure S1D.

P16^INK4A^ levels have been proposed to cause CDK4/6 inhibitor resistance in different cancer types, but only limited information is available for AML. To investigate INK4 protein levels in AML, we analysed microarray data from healthy human bone marrow (BM) and CDK6-dependent AML subtypes harbouring RUNX1-RUNX1T1 t(8;21) fusion or KMT2A rearrangements t(11q23) (KMT2Ar) with a focus on KMT2A-MLLT3 t(9;11). RUNX1-RUNX1T1 and KMT2A-MLLT3 are among the most common karyotype aberrations in AML [28,29]. KMT2A-MLLT3+ AML patients display significantly increased *CDKN2A* (p16^INK4A^) expression compared to healthy BM and RUNX1-RUNX1T1+ patients (Figure 1A). No alterations of *CDKN2B* (p15^INK4B^) expression were detected. A significant reduction in *CDKN2C* (p18^INK4C^) was present in KMT2Ar patients compared to RUNX1-RUNX1T1+ and KMT2A-MLLT3+ patients. Compared to healthy BM, the reduction was not significant. Notably, the KMT2A-MLLT3+ patient samples were separated into two populations: one group of samples showed increased *CDKN2C* (p18^INK4C^) expression compared to healthy BM and the other AML subtypes, whereas a distinct subset of patients exhibited low *CDKN2C* (p18^INK4C^) levels. *CDKN2C* (p18^INK4C^) and *CDKN2A* (p16^INK4A^) showed a significant positive correlation, which is indicative of a coregulation (Figure S1A). *CDKN2D* (p19^INK4D^) expression was significantly reduced in RUNX1-RUNX1T1+ patient samples compared to all other groups (Figure 1A).

To gain further insights, we took advantage of a recently established cellular model. We generated murine leukemic progenitor cell lines (HPC^LSK^) [21], into which we retrovirally introduced RUNX1-RUNX1T1 or KMT2A-MLLT3. Individual HPC^LSK^ AML cells were then derived (Figure 1B). The transformed HPC^LSK^ cells were able to induce disease in vivo [21]. This system enabled the concomitant investigation of the oncogene induced effects in genetically comparable parental cells. Flow cytometry analysis and mRNA expression (Figure S1B,C) verified the presence of the oncogenic fusion proteins that harbour an IRES Venus (KMT2A-MLLT3) or an IRES GFP (RUNX1-RUNX1T1). Four distinct clones of each AML subtype were used for further studies.

Using an unpublished RNA sequencing dataset, we focused on the INK4 expressions of the HPC^LSK^ AML lines compared to the maternal untransformed HPC^LSK^ (WT) lines (Figure 1C). *Cdkn2c* (p18^INK4C^) was consistently expressed at high levels in KMT2A-MLLT3+ cells compared to RUNX1-RUNX1T1+ and control cells. In line with the situation in human patients, we observed a trend of increased *Cdkn2a* (p16^INK4A^) expression in both oncogenic settings compared to the controls. *Cdkn2d* (p19^INK4D^) and *Cdkn2b* (p15^INK4B^) did not show any significant differences between transformed and untransformed cells.

The high *Cdkn2c* (p18^INK4C^) mRNA levels in the KMT2A-MLLT3+ HPC^LSK^ cells compared to the RUNX1-RUNX1T1+ HPC^LSK^ cells were verified at protein level by immunoblot analysis (Figure 1D, Figure S1D and the original immunoblots in Figure S5). P16^INK4A^ protein levels were higher in KMT2A-MLLT3+ compared to RUNX1-RUNX1T1+ samples, pointing to the involvement of translational regulation. Notably, a single KMT2A-MLLT3+ HPC^LSK^ cell line displayed low p16^INK4A^ and p18^INK4C^ protein levels compared to the RUNX1-RUNX1T1+ cell lines. This cell line also showed low RNA expression of *Cdkn2a* (p16^INK4A^) and *Cdkn2c* (p18^INK4C^), resembling the KMT2A-MLLT3+ patient samples with low *CDKN2A* (p16^INK4A^) and *CDKN2C* (p18^INK4C^) levels (Figure S1E,F).

These data led us to conclude that human and murine RUNX1-RUNX1T1+ and KMT2A-MLLT3+ transformed cells are characterised by distinct INK4 expression patterns on RNA and protein levels.

### 3.2. Cells with Low INK4 Levels Show Fast Cell Cycle Re-Entry after BSJ Treatment

Low p16^INK4A^ levels are associated with high sensitivity to palbociclib, which induces cell cycle arrest in several human cancer cells of distinct origins, including lung and gastric cancer cell lines [30]. The impact of INK4 proteins on CDK6 degrader treatment response has not been explored. The murine HPC^LSK^ AML cell lines reflected the situation of p16^INK4A^ and p18^INK4C^ expression in human AML and were therefore used as the model system.

We first tested different treatment conditions using various concentrations and time points of the CDK6 degrader BSJ in the HPC^LSK^ AML lines [3] (Figure S2A–D, uncropped immunoblots in Figure S5). Irrespective of the BSJ concentrations that were used, we failed to detect a complete CDK6 degradation (Figure S2A,B). We found a CDK6 reduction of 80% after 240 min of BSJ treatment using 3 µM (Figure S2C,D). CDK4 was not affected under these conditions (Figure S2B, right-hand panel).

**Figure 2:**
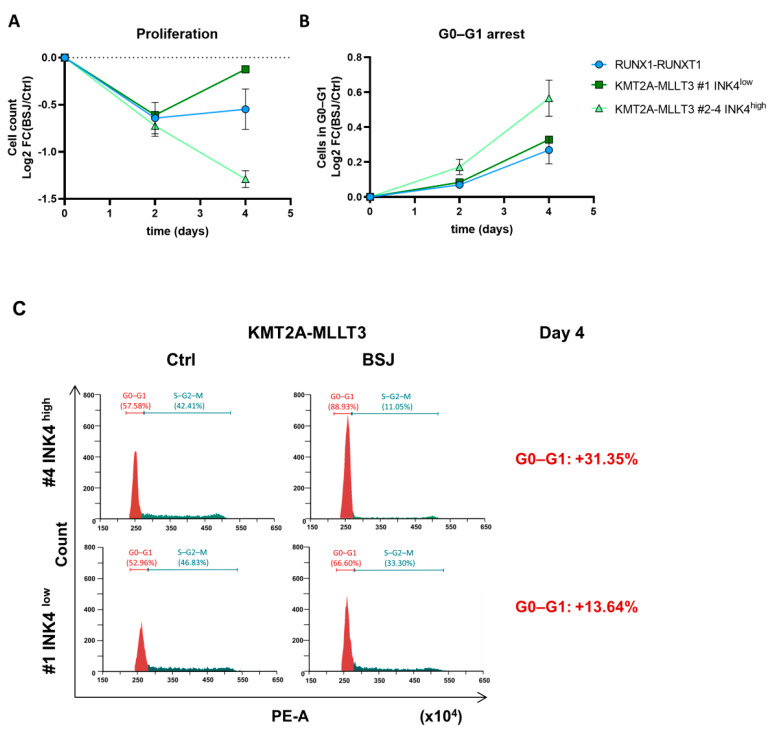
Delayed cell cycle re-entry in AML cells with high INK4 levels after BSJ treatment. (**A**) The flow cytometry analysis of RUNX1-RUNX1T1+ (*n* = 3), KMT2A-MLLT3+ INK4^low^ (*n* = 1) and INK4^high^ (*n* = 3) cells. The cell number measurements are depicted as the log2 FC (BSJ/control) calculation after two and four days of treatment with 3 µM of BSJ. (**B**) The flow cytometry analysis of cell cycle phases using propidium iodide (PI). The G0–G1 phase of RUNX1-RUNX1T1+ (*n* = 3), KMT2A-MLLT3+ INK4^low^ (*n* = 1) and INK4^high^ (*n* = 3) cells is depicted as the log2 FC (BSJ/control) calculation after two and four days of treatment with 3 µM of BSJ. (**C**) The exemplary cell cycle profiles that were analysed by the PI staining of KMT2A-MLLT3+ #4 INK4^high^ (**top** panel) and #1 INK4^low^ cells (**bottom** panel) at day four of treatment with 3 µM of BSJ (right-hand panel) or vehicle control (**left**-hand panel). The gating strategy is indicated in the histogram plots. The change of the percentage of cells in the G0–G1 phase after BSJ treatment is highlighted on the right-hand side of the graphs.

We analysed the proliferation and cell cycle phases of the BSJ treated KMT2A-MLLT3+ and RUNX1-RUNX1T1+ HPC^LSK^ cells at several time points. Cell numbers significantly decreased in KMT2A-MLLT3+ cells on days two and four of BSJ treatment (Figure S2E). When investigating the individual KMT2A-MLLT3+ cell lines, we realised that the INK4^low^ cells overcame the proliferation arrest after four days, which was not the case in cell lines with high INK4 levels (Figure 2A). RUNX1-RUNX1T1+ cells showed reduced cell numbers following CDK6 degradation, although the data did not reach levels of significance (Day 2: *p* = 0.06). Similar to the KMT2A-MLLT3+ INK4^low^ cells, the RUNX1-RUNX1T1+ cells overcame proliferation arrest faster (Figure 2A). Similar to previous studies with CDK4/6 inhibitors [6], we also observed a significant increase in myeloid markers following CDK6 degradation (Figure S2F). No significant differences in cell viability were detected (Figure S2G). This led us to conclude that reduced proliferation was not a result of apoptosis but of cell cycle arrest and cell differentiation. Cells in G0–G1 phase were significantly decreased in KMT2A-MLLT3+ and RUNX1-RUNX1T1+ cells after two days of BSJ treatment (Figure 2B and Figure S2H, left-hand panels). After four days, only KMT2A-MLLT3+ cells still remained in a significant G0–G1 arrest. RUNX1-RUNX1T1+ cells and KMT2A-MLLT3+ INK4^low^ cells had already started to enter the cell cycle, as indicated by the decreased percentage of cells in the G0–G1 phase after four days of BSJ treatment (Figure 2B and Figure S2H, right-hand panels). The exemplary flow cytometry plots of KMT2A-MLLT3+ #1 INK4^low^ and #4 INK4^high^ cells show that the percentage of cells in G0–G1 with high INK4 levels were increased compared to cells with low INK4 levels (Figure 2C and Figure S2I). Combined treatment with the CDK6 degrader and the CDK4/6 inhibitor palbociclib (PD-0332991) indicated at least a partial cell cycle regulation by CDK4 following CDK6 degradation in the INK4^low^ cells (Figure S2J).

Taken together, these data highlight that RUNX1-RUNX1T1+ cells, as well as the KMT2A-MLLT3+ INK4^low^ line, exhibited a decreased proliferation arrest after CDK6-targeted therapy compared to the KMT2A-MLLT3+ INK4^high^ cells.

### 3.3. BSJ Treatment Enriches CDK6-INK4 Complexes

**Figure 3:**
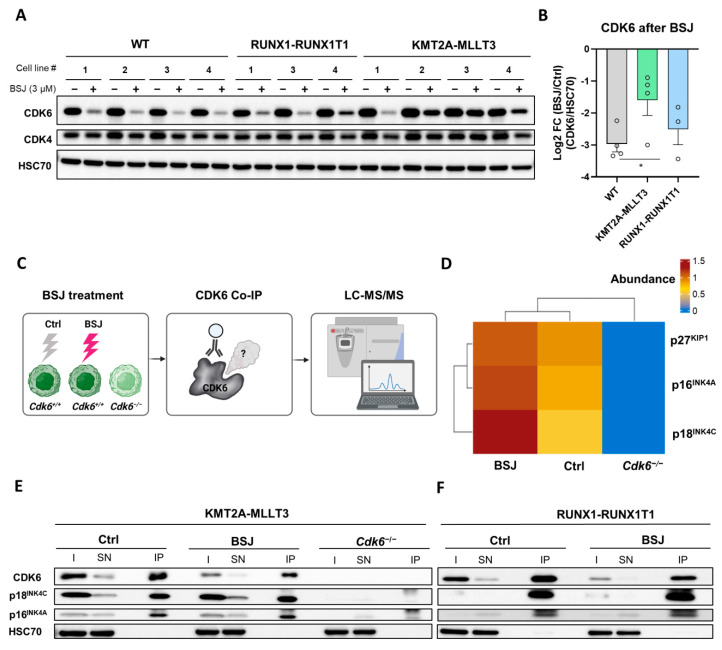
CDK6-INK4 complexes were enriched after BSJ treatment. (**A**) The immunoblot for the CDK6 and CDK4 levels of HPC^LSK^ WT (*n* = 4), RUNX1-RUNX1T1+ (*n* = 3) and KMT2A-MLLT3+ (*n* = 4) cells treated with 3 µM of BSJ or vehicle control for four days. HSC70 served as a loading control. The original, uncropped immunoblots are depicted in Figure S5. The densitometry is depicted in (**B**) and Figure S3A. (**B**) The log2FC (BSJ/control) calculation of the immunoblot densitometry quantification from (**A**) (CDK6/HSC70): * *p* < 0.05. (**C**) A schematic representation of the experimental procedure. KMT2A-MLLT3+ cells were treated with 3 µM of BSJ or vehicle control for 24 h. A KMT2A-MLLT3+ *Cdk6^−/−^* cell line was used as the negative control. Cell protein lysates were co-immunoprecipitated (Co-IP) with an anti-CDK6 antibody and complexes were analysed by LC-MS/MS to identify any CDK6 interaction partners that were present in the untreated sample or were enriched after BSJ treatment. (**D**) A heat map depicting the abundances of INK4 and KIP1 proteins in the BSJ and control samples. The enrichments of the proteins over the background were calculated as the log2 fold changes (log2FC) of the normalised abundances of the control or BSJ over the *Cdk6^−/−^*. (**E**) An anti-CDK6 Co-IP was performed on KMT2A-MLLT3+ cell lysates from 24 h treatments with 3 µM of BSJ or vehicle control. *Cdk6^−/−^* cells served as the negative control. The input (I), supernatant (SN) and immunoprecipitate (IP) fractions were immunoblotted for CD K6, p18^INK4C^ and p16^INK4A^. HSC70 served as the loading control. The immunoblot densitometry quantification is depicted in Figure S3F. The original, uncropped immunoblots are depicted in Figure S5. (**F**) An anti-CDK6 Co-IP was performed on RUNX1-RUNX1T1+ cell lysates from 24 h treatments with 3 µM of BSJ or vehicle control. The input (I), supernatant (SN) and immunoprecipitate (IP) fractions were immunoblotted for CDK6, p18^INK4C^ and p16^INK4A^. HSC70 served as the loading control. The immunoblot densitometry quantification is depicted in Figure S3G. The original, uncropped immunoblots are depicted in Figure S5.

To investigate the mechanism accounting for the distinct treatment outcomes between AML subtypes, we analysed degradation efficacy in HPC^LSK^ WT and AML lines. AML subtypes reacted differently to BSJ treatment (Figure 3A,B and Figure S3A and the original immunoblots in Figure S5). BSJ induced significantly less CDK6 degradation in KMT2A-MLLT3+ (mean log2 FC (BSJ/control) of −1.60) compared to WT cells (mean log2 FC (BSJ/control) of −2.97) (Figure 3A,B). Treatment with low concentrations already resulted in different outcomes between KMT2A-MLLT3+ and RUNX1-RUNX1T1+ cells (Figure S3B,C and original immunoblots in Figure S5). At 0.75 µM of BSJ, the RUNX1-RUNX1T1+ cells (mean log2 fold change (FC) (BSJ/control) of −1.34) possessed significantly less remaining CDK6 compared to KMT2A-MLLT3+ cells (mean log2 FC (BSJ/control) of −0.61) (Figure S3C). These findings show that BSJ efficacy varied among the AML subtypes and provoked the question of whether high INK4 levels account for the reduced degradation efficacy.

This hypothesis was supported by a study in breast cancer cells that proposed the role of INK4 proteins in palbociclib binding to CDK4/6 [31]. As BSJ was developed based on the structure of palbociclib, the binding site on CDK6 is preserved [3]. We thus investigated whether INK4–CDK6 complexes are resistant to BSJ degradation. We decided to immunoprecipitate (IP) CDK6 complexes in the absence and presence of BSJ and to subject the complexes to LC-MS/MS analysis. This analysis allowed for the identification of potential differential CDK6 protein interaction partners that could confer resistance to BSJ treatment. We used a KMT2A-MLLT3+ cell line with high INK4 levels and treated it with BSJ or the vehicle control. KMT2A-MLLT3 *Cdk6*^−/−^ cells served as an additional background negative control to remove any unspecific proteins from the experiment [32] (Figure 3C). The LC-MS/MS data provided a list of proteins that were selectively enriched in the untreated sample (218; 20.9%), restricted to the BSJ treated sample (293; 28%) or present under both conditions (534; 51.1%) (Figure S3D). The BSJ treatment enriched the two INK4 proteins p16^INK4A^ and p18^INK4C^ and, to a lesser extent, p27^KIP1^ (Figure 3D and Figure S3E). We validated these findings in two cell lines, expressing RUNX1-RUNX1T1 or KMT2A-MLLT3 by performing a CDK6 IP followed by immunoblotting for p16^INK4A^ and p18^INK4C^. As expected, substantially less CDK6 was immunoprecipitated after the BSJ treatment (Figure 3E,F and the original, uncropped immunoblots are depicted in Figure S5 and the densitometry is presented in Figure S3F,G). The BSJ treatment did not reduce the levels of p16^INK4A^ and p18^INK4C^ that were coimmunoprecipitated with CDK6, which is indicative of these complexes being protected from degradation.

These findings show that p16^INK4A^ and p18^INK4C^ proteins interfere with BSJ-mediated CDK6 degradation.

### 3.4. INK4 Levels Predict BSJ-Mediated CDK6 Degradation Efficacy in Murine and Human Leukaemia

Previous research of Philadelphia chromosome–positive acute lymphoblastic leukaemia (Ph+ ALL) using a CDK6 degrader showed the rapid and preferential degradation of CDK6 over CDK4 [20]. Degrader treatment resulted in proliferation inhibition and suppressed patient-derived Ph+ ALL formation in mice. We used this leukemic cell type to validate our findings in a genetically engineered BCR-ABL^p185+^ lymphoid model (Figure S4A,B and the original immunoblots in Figure S5). P16^INK4A^ is one of the most commonly altered genes in leukaemia and therefore, represents a crucial biomarker. *Cdkn2a^−/−^* and *Cdk6^−/−^* BCR-ABL^p185+^ cells with and without the re-expression of *Cdkn2a* and *Cdk6* were treated with BSJ, and the degradation efficacy was compared between *Cdkn2a* expressing and non-expressing cells. Again, the cells lacking p16^INK4A^ showed a more effective CDK6 degradation compared to the control cells expressing p16^INK4A^, mimicking the data obtained from the KMT2A-MLLT3+ HPC^LSK^ cells.

**Figure 4:**
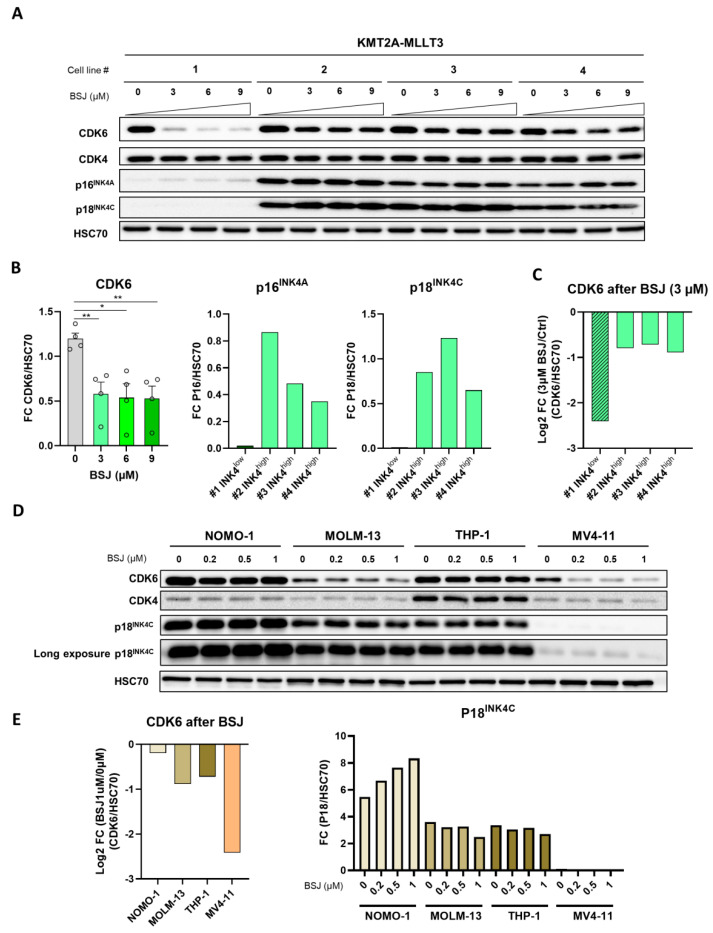
BSJ efficacy predicted by INK4 levels. (**A**) The immunoblot for CDK6, CDK4, p16^INK4A^, p18^INK4C^ and HSC70 in KMT2A-MLLT3+ cells (*n* = 4) treated with increasing concentrations (3 µM, 6 µM and 9 µM) of BSJ or vehicle control for four days. The original, uncropped immunoblots are depicted in Figure S5. The densitometry quantification is depicted in (**B**,**C**) and Figure S4C,D. (**B**) The immunoblot densitometry quantification from (**A**) of CDK6, p16^INK4A^ and p18^INK4C^ normalised to HSC70 (**left**) and P16^INK4A^ and p18^INK4C^ quantification normalised to the HSC70 of KMT2A-MLLT3 control samples grouped into INK4^low^ and INK4^high^ (**middle** and **right**): * *p* < 0.05; ** *p* < 0.01. The densitometry of CDK4 is depicted in Figure S4D. (**C**) Log2 FC (BSJ/control) calculated from the CDK6/HSC70 immunoblot quantification from the KMT2A-MLLT3+ samples grouped into INK4^low^ and INK4^high^. (**D**) The immunoblot for CDK6, CDK4 and p18^INK4C^ and the short and long exposure and HSC70 of human AML cell lines that were positive for KMT2A-MLLT3 (NOMO-1, MOLM-13 and THP-1) or KMT2A-AFF1 (MV4-11) and were treated with increasing concentrations of BSJ (0.2 µM, 0.5 µM and 1 µM) or vehicle control for 90 min. The original, uncropped immunoblots are depicted in Figure S5. The densitometry quantification is depicted in (**E**) and Figure S4E,F. (**E**) Log2 FC (BSJ/control) of the immunoblot densitometry quantification from (**D**) calculated from the human AML cell lines that were treated with 1 µM of BSJ (**left**). The immunoblot quantification of the p18^INK4C^ levels normalised to HSC70 from the human AML cell lines that were treated with increasing concentrations of BSJ (0.2 µM, 0.5 µM and 1 µM) or vehicle control for 90 min and the densitometry for CDK4 and CDK6 is depicted in Figure S4E.

To further link CDK6 degradation efficacy to INK4 proteins, we utilised KMT2A-MLLT3+ transformed HPC^LSK^ cell lines. We compared the BSJ efficacies of KMT2A-MLLT3+ INK4^low^ cells to the three KMT2A-MLLT3 INK4^high^ cell lines (Figure 4A–C and the original uncropped immunoblots in Figure S5). Indeed, the KMT2A-MLLT3+ cell line that degraded CDK6 most potently after BSJ treatment was the #1 INK4^low^ cell line (log2 FC (BSJ/control) (−2.41)) (Figure 4C). A significantly reduced CDK6 degradation was observed in the presence of high p16^INK4A^ and p18^INK4C^ levels (log2 FC (BSJ/control) #2 (−0.80), #3 (−0.71) and #4 (−0.89)), despite increased BSJ concentrations of up to 9 µM (Figure S4C). CDK4 was only degraded at high concentrations of BSJ, although to a minor degree (Figure S4D; top left-hand panel).

To extend our findings to a human setting, we treated human AML cell lines harbouring KMT2A rearrangements with different concentrations of BSJ. We used the KMT2A-MLLT3+ cell lines NOMO-1, THP-1 and MOLM-13 and the KMT2A-AFF1+ cell line MV4-11 (Figure 4D,E, Figure S4E,F and the original immunoblots in Figure S5). In line with our previous results, the MV4-11 cell line displayed high CDK6 degradation efficacy and had low p18^INK4C^ protein levels. The observation that the three KMT2A-MLLT3+ cell lines displayed higher p18^INK4C^ levels than the KMT2A-AFF1+ cell line supports the human RNA sequencing data showing that AML patient cells harbouring KMT2A-MLLT3 have higher *CDKN2C* p18^INK4C^ expression than the pool of different KMT2A rearranged AML patient cells (Figure 1A). P16^INK4A^ could not be detected in these cell lines (data not shown), letting us conclude that p18^INK4C^ is the major cause of the reduced CDK6 degradation in human AML cell lines.

These data unequivocally link p16^INK4A^ and p18^INK4C^ protein levels to the efficacy of BSJ-mediated CDK6 degradation in human and murine leukemic cells.

## 4. Discussion

CDK6 is a key regulator of several AML subtypes, making it a favourable therapeutic target for this aggressive haematologic disease. CDK4/6 kinase inhibitors are in clinical use and CDK6 degraders represent a therapeutic alternative and bear the advantage of blocking kinase-dependent and kinase-independent functions. Kinase-independent functions are of particular relevance in HSCs and LSCs.

Here, we showed that successful pharmacological CDK6 degradation in AML depends on INK4 protein levels. Given the great heterogeneity among AML subtypes, it is crucial to study INK4 expression in specific oncogenic settings as we clearly observed oncogene-dependent differences in INK4 levels. We found that INKs provide resistance towards CDK6 degradation in human and murine RUNX1-RUNX1T1+ and KMT2A rearranged cells.

We observed two distinct populations with high and low *CDKN2C* expressions in the human AML datasets of KMT2A-MLLT3+ patients. The different *CDKN2C* expressions positively correlate with *CDKN2A* expression, suggesting the coregulation of these two INK4 genes. In line with the human data, a clear increase in p16^INK4A^ and p18^INK4C^ expression in three of the four murine KMT2A-MLLT3+ HPC^LSK^ cell lines was detected. Elevated p18^INK4C^ expression is in line with literature showing that KMT2A-MLLT3 directly binds to the *CDKN2C* locus, thereby driving its expression [33], but an active downregulation of *CDKN2C* by epigenetic mechanisms has not yet been reported. Further investigations are needed to understand the mechanisms of this coregulation and whether this is related to epigenetic modulations, cell differentiation or other cellular states and conditions.

We identified p16^INK4A^ and p18^INK4C^ as major CDK6 binding partners that block BSJ-mediated CDK6 degradation. Previous studies in breast cancer have indicated the contribution of INK4 proteins to palbociclib sensitivity [13,14]. A recent study has suggested that CDK4/6 degraders are promising alternatives to CDK4/6 kinase inhibitors as they suppress the proliferation of palbociclib resistant breast cancer cells [31]. However, the impact of INK4 proteins on CDK6 degradation efficacy has not been shown. This study demonstrated that INK4 proteins compete with palbociclib for the same CDK6 binding site and that CDK6–INK4 complexes are still able to bind to ATP, thereby driving CDK4/6 kinase functions.

We investigated BSJ efficacy in cells with high expressions of INK4 protein levels. As the binding site of the degrader BSJ to CDK6 was designed based on the molecular structure of palbociclib [3], we hypothesised that CDK6–INK4 complexes are un-targetable by the CDK6 degrader. We verified this hypothesis by comparing murine and human AML cell lines with high or low expressions of p16^INK4A^ and/or p18^INK4C^ and murine *Cdkn2a^−/−^* vs. *Cdkn2a^+/+^* BCR-ABL^p185+^ cells modelling acute lymphoblastic leukaemia (ALL). In all of the examined in vitro models, we clearly observed reduced BSJ efficacy in the presence of high p16^INK4A^ and/or p18^INK4C^ protein levels. We monitored the accelerated cell cycle re-entry of the KMT2A-MLLT3 #1 INK4^low^ cell line and the low INK4 RUNX1-RUNX1T1 cells. This dataset is supported by literature indicating that high p16^INK4A^ levels confer palbociclib resistance; however, low p16^INK4A^ levels do not necessarily induce high palbociclib sensitivity [13]. One possible scenario could be that CDK4 takes over cell cycle regulation, which is normally controlled by CDK6 in cells with low INK4 levels. In cells with high INK4 levels, CDK4 is not able to compensate for the loss of CDK6 as it is inhibited by the INK4 proteins. On the other hand, it has also been proposed that palbociclib resistance can occur as a result of the escape mechanisms of the cell cycle machinery that could be activated, such as the CDK2–cyclin A/E axis [34,35]. These facts indicate that the combined treatment of a CDK6 degrader together with inhibitors of CDK2 and CDK4 may hold promising results and should be considered for future investigations, specifically for INK4^low^ cancer cells.

Experiments in breast cancer cells suggest that CDK6 fulfils kinase-dependent roles despite p18^INK4C^ binding. As it was shown that CDK6 also confers transcriptional regulation in a kinase-dependent and kinase-independent manner [19], it is of particular interest to discover whether the CDK6 remaining after BSJ treatment is still involved in these mechanisms. The role of INK4 binding for CDK6-dependent transcriptional regulation is still unsolved. We can speculate that CDK6–INK4 complexes harbour specific functions that are unrelated to cell cycle regulation, leading to different therapeutic outcomes between INK4^high^ and INK4^low^ cells. Small molecule compounds inhibiting p18^INK4C^ have been developed and may aid in addressing these issues [36,37,38].

Our findings highlight that INK4 proteins are not only differentially expressed among AML subtypes but also contribute to CDK6-targeted therapeutic efficacy.Therefore, they can be considered as predictive indicators of treatment success and guide further strategies. Moreover, our data show that novel CDK6-specific therapies should be established to target CDK6 sites, which do not compete with INK4 or CIP/KIP binding to overcome therapeutic limitations.

## 5. Conclusions

CDK6-specific degraders are a promising tool for the targeted therapy of AML, based on the high dependency of AML cells on CDK6. Understanding their mechanisms of action is a prerequisite for their optimal therapeutic use. We show that the levels of INK4 protein family members p16^INK4A^ and p18^INK4C^ are critical determinants of CDK6 degradation. After INK4 binding, CDK6 is not targeted by the CDK6 degrader. These results highlight the dependency of CDK6 degradation on its interaction partners. We propose to develop novel treatment strategies that avoid interference with INK4 binding sites.

## Data Availability

The data presented in this study are available within the Supplementary Material or on request from the corresponding author. The mass spectrometry proteomics data have been deposited to the ProteomeXchange Consortium via the PRIDE [39] partner repository with the dataset identifier PXD032216.

## Supplementary Materials

The following supporting information can be downloaded at: https://www.mdpi.com/article/10.3390/cancers14061554/s1, Figure S1: Expression of INK4 proteins in AML cells harbouring KMT2A-MLLT3 or RUNX1-RUNX1T1, Figure S2: Accelerated cell cycle re-entry of INK4^low^ cells, Figure S3: BSJ efficacy depends on INK4 protein levels, Figure S4: INK4 levels as predictive markers for CDK6 degrader, Figure S5: Original uncropped immunoblot scans, Figure S6: Plasmid maps, Supplementary Methods, Table S1: Primer sequences for qPCRs, Table S2: Antibodies used in this study. And a detailed method description of liquid chromatography coupled with tandem mass spectrometry (LC-MS/MS) [39,40,41,42], Table S3: Raw and normalised mass spectrometry (LC-MS/MS) data.
