# Supplementary material for: CDK6 Degradation Is Counteracted by p16INK4A and p18INK4C in AML
Source: Cancers (Basel). 2022 Mar 18;14(6):1554. doi: 10.3390/cancers14061554 (PMC8946512; doi:10.3390/cancers14061554)
Supplement: Supplementary file 1 [file cancers-14-01554-s001.zip › cancers-1605912-SI.pdf]

Supplementary Materials

CDK6 Degradation is Counteracted by p16^{INK4A} and p18^{INK4C} in AML

Belinda S. Schmalzbauer [1], Teresemary Thondanpallil [1], Gerwin Heller [2], Alessia Schirripa [1], Clio-Melina Sperl [1], Isabella M. Mayer [1], Vanessa M. Knab [1], Sofie Nebenfuehr [1], Markus Zojer [1], André C. Mueller [3], Frédéric Fontaine [3], Thorsten Klampfl [1], Veronika Sexl [1] and Karoline Kollmann [1,*]

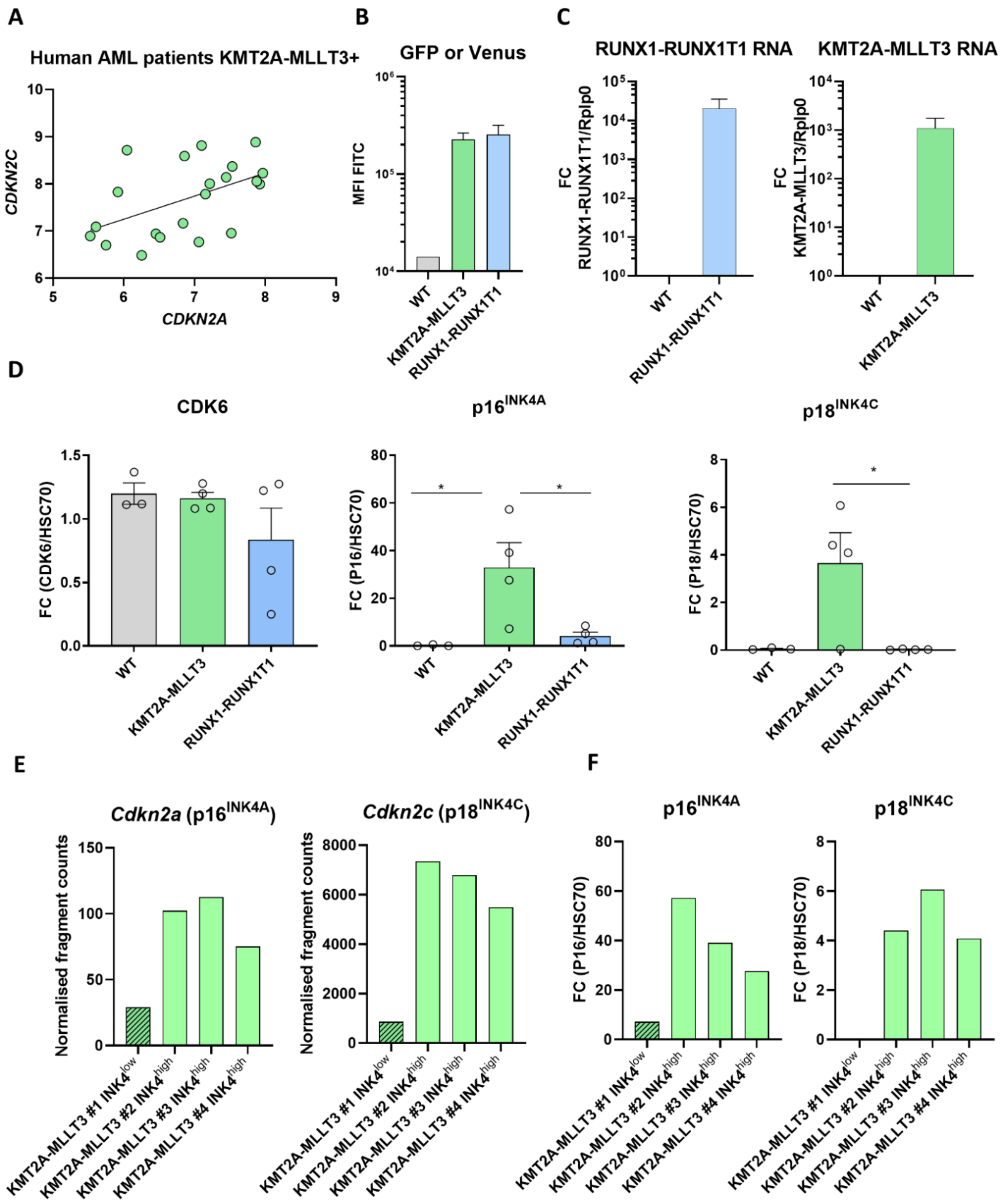

Supplementary Materials

Figure S1. Expression of INK4 proteins in AML cells harboring KMT2A-MLLT3 or RUNX1-RUNX1T1**.** **(A)** Correlation analysis from human AML patients harboring KMT2A-MLLT3. Depicted are RNA expressions of *CDKN2A* and *CDKN2C.* (Pearson R=0.5002, *p*=0.02). **(B)** Flow cytometry analysis of HPC^{LSK} AML cells expressing Venus (KMT2A-MLLT3) (n=4) and GFP (RUNX1-RUNX1T1) (n=4) detected in the FITC channel and compared to WT (n=1) as negative control. **(C)** qPCR analysis of overexpressed human oncogenes RUNX1-RUNX1T1 (n=3) and KMT2A-MLLT3 (n=4) in the HPC^{LSK} cells compared to WT (n=1) as negative control. **(D)** Immunoblot quantification of HPC^{LSK} WT (n=3), KMT2A-MLLT3+ (n=4) and RUNX1-RUNX1T1+ (n=4) cells on CDK6, $p16^{INK4A}$ and $p18^{INK4C}$. **(E)** RNA expressions taken from an extensive, unpublished RNA Seq data set of *Cdkn2a* and *Cdkn2c* in the HPC^{LSK} KMT2A-MLLT3+ lines (n=4) grouped into $INK4^{high}$ (n=3) and $INK4^{low}$ (n=1). **(F)** Immunoblot quantifications of $p16^{INK4A}$ and $p18^{INK4C}$ in the HPC^{LSK} KMT2A-MLLT3+ (n=4) lines, grouped into $INK4^{high}$ and $INK4^{low}$ cells.

Supplementary Materials

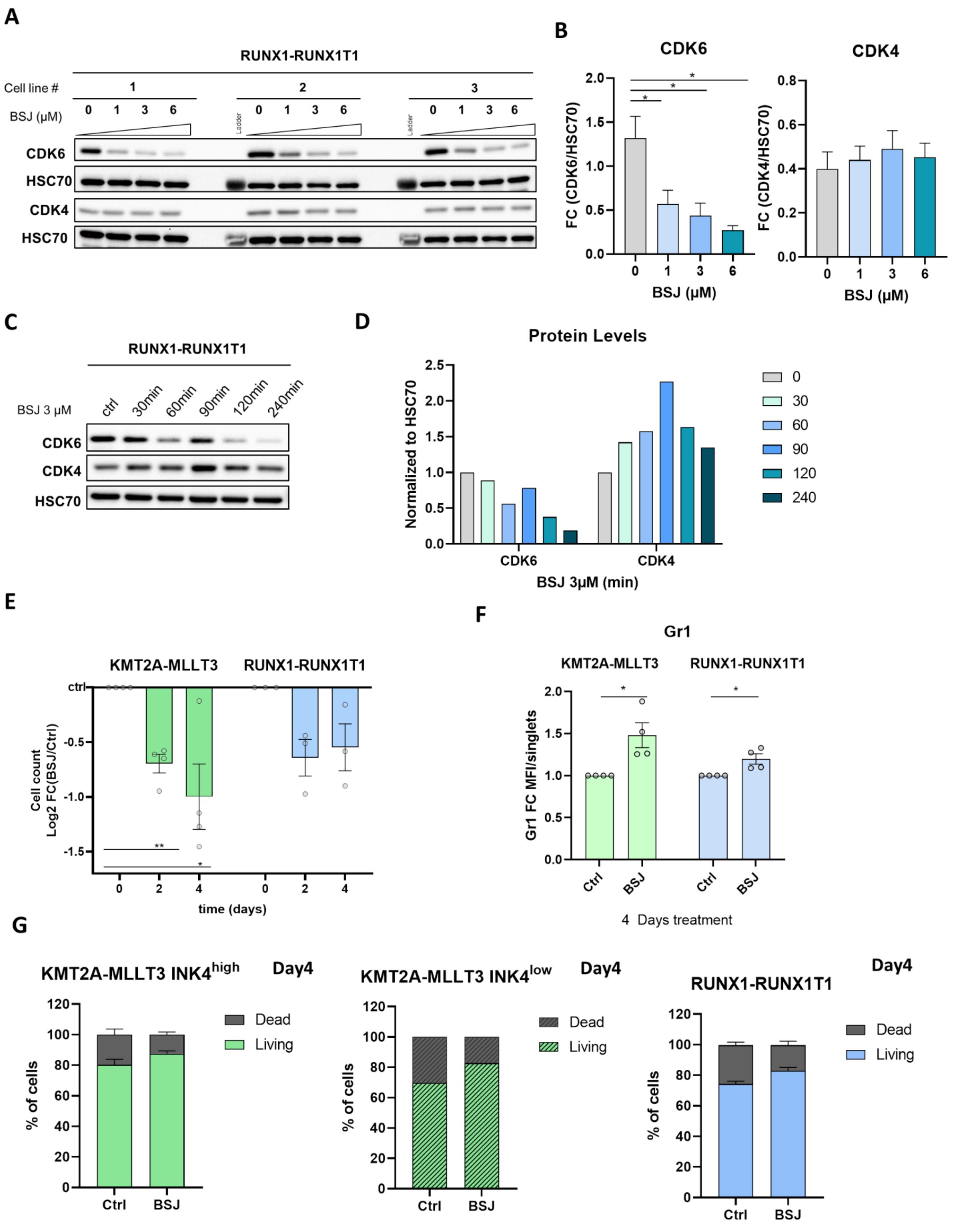

H

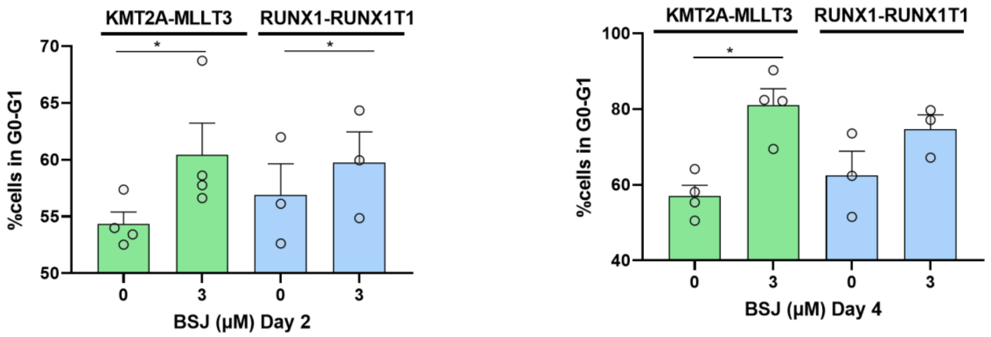

I

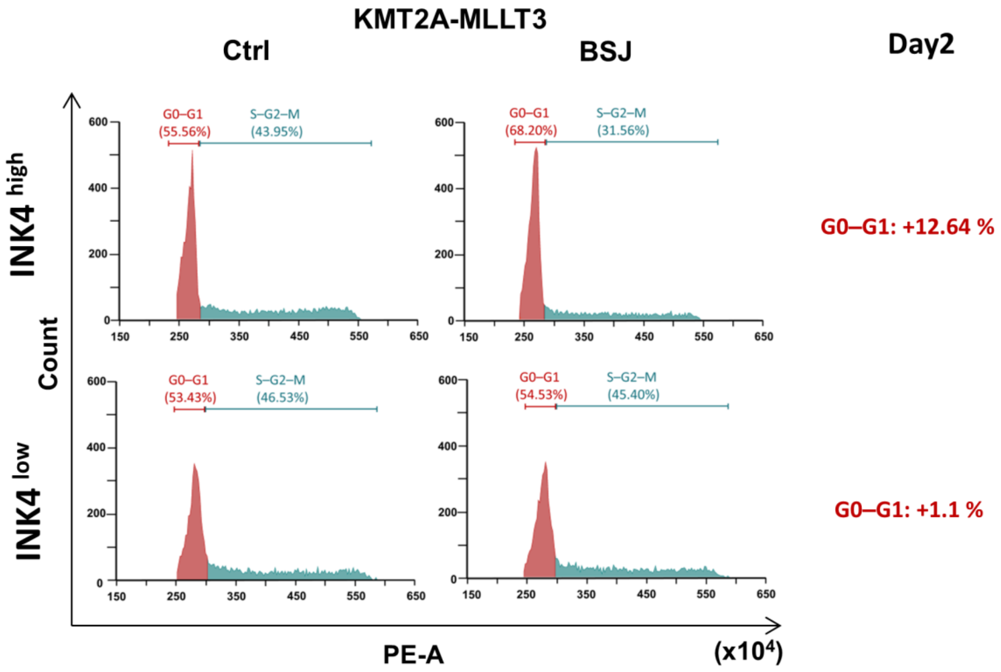

J

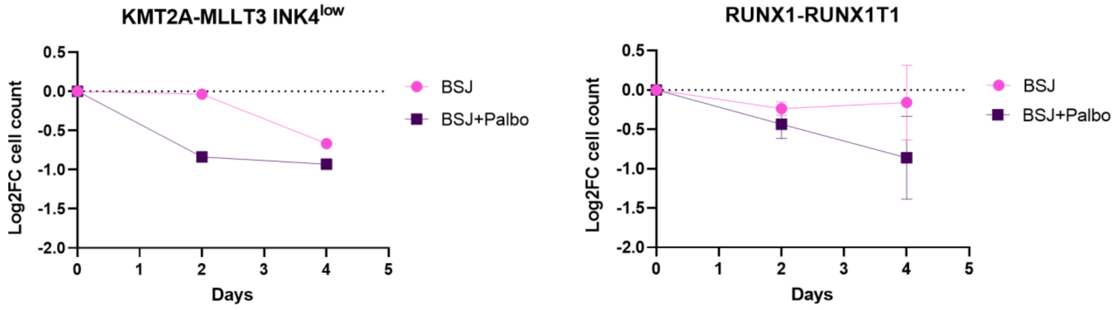

Figure S2. Accelerated cell cycle re-entry of INK4[low] cells. **(A)** Immunoblot for CDK6, CDK4 and HSC70 of RUNX1-RUNX1T1+ cells (n=3) treated with increasing concentrations of BSJ (1 µM, 3 µM and 6 µM) or vehicle control for 4 days. **(B)** Immunoblot quantification of (A) from CDK6 and CDK4 normalized to HSC70 (bottom). *p <0.05. **(C)** Immunoblot for CDK6, CDK4 and HSC70 from RUNX1-RUNX1T1+ cell lysates of treatments with 3 µM BSJ for indicated time points (30, 60, 90, 120 and 240 min). Vehicle treated sample (Ctrl) was harvested after 240 min. **(D)** Immunoblot quantification of (C) from CDK4 and CDK6 normalized to respective HSC70 control. **(E)** Flow cytometry

analysis of RUNX1-RUNX1T1+ (n=3) KMT2A-MLLT3+ (n=4) cells measuring cell numbers as Log2 FC (BSJ/Ctrl) calculation after two and four days of treatment with 3 μM BSJ or vehicle control. **p* = 0.0442; ***p* =0.0038. **(F)** BSJ induces myeloid differentiation. Cells were treated four days with 3 μM BSJ and expression of Gr1 was measured by flow cytometry. **p* <0.05 **(G)** Flow cytometry analysis of cell viability using propidium iodide (PI) of KMT2A-MLLT3+ $INK4^{high}$ (n=3; top panel), KMT2A-MLLT3 $INK4^{low}$ (n=1; middle panel) and RUNX1-RUNX1T1+ (n=3; bottom panel) cells after four days of treatment with 3 μM BSJ or vehicle control. **(H)** Flow cytometry analysis of G0−G1 cell cycle phases using PI of RUNX1-RUNX1T1+ (n=3) KMT2A-MLLT3+ (n=4) cells after two (left panel) and four (right panel) days of treatment with 3 μM BSJ or vehicle control.**p* < 0.05. **(I)** Exemplary cell-cycle profiles analyzed by PI-staining of KMT2A-MLLT3+ #4 $INK4^{high}$ (top panel) and #1 $INK4^{low}$ (bottom panel) cells at day two of treatment with 3 μM BSJ (right panel) or vehicle control (left panel). The gating strategy is indicated in the histogram plots. The change of percentage of cells in G0−G1 after BSJ treatment is highlighted on the right of the graphs**. (J)** BSJ+Palbociclib (Palbo) combination to analyze the contribution of CDK4. Cells were treated with BSJ for 24 h followed by addition of the CDK4/6 inhibitor Palbociclib on Day 1. KMT2A-MLLT3 cells (left panel) and RUNX1-RUNX1T1 cells (right panel) were analyzed over the course of four days. Cell number was measured via flow cytometry.

Supplementary Materials

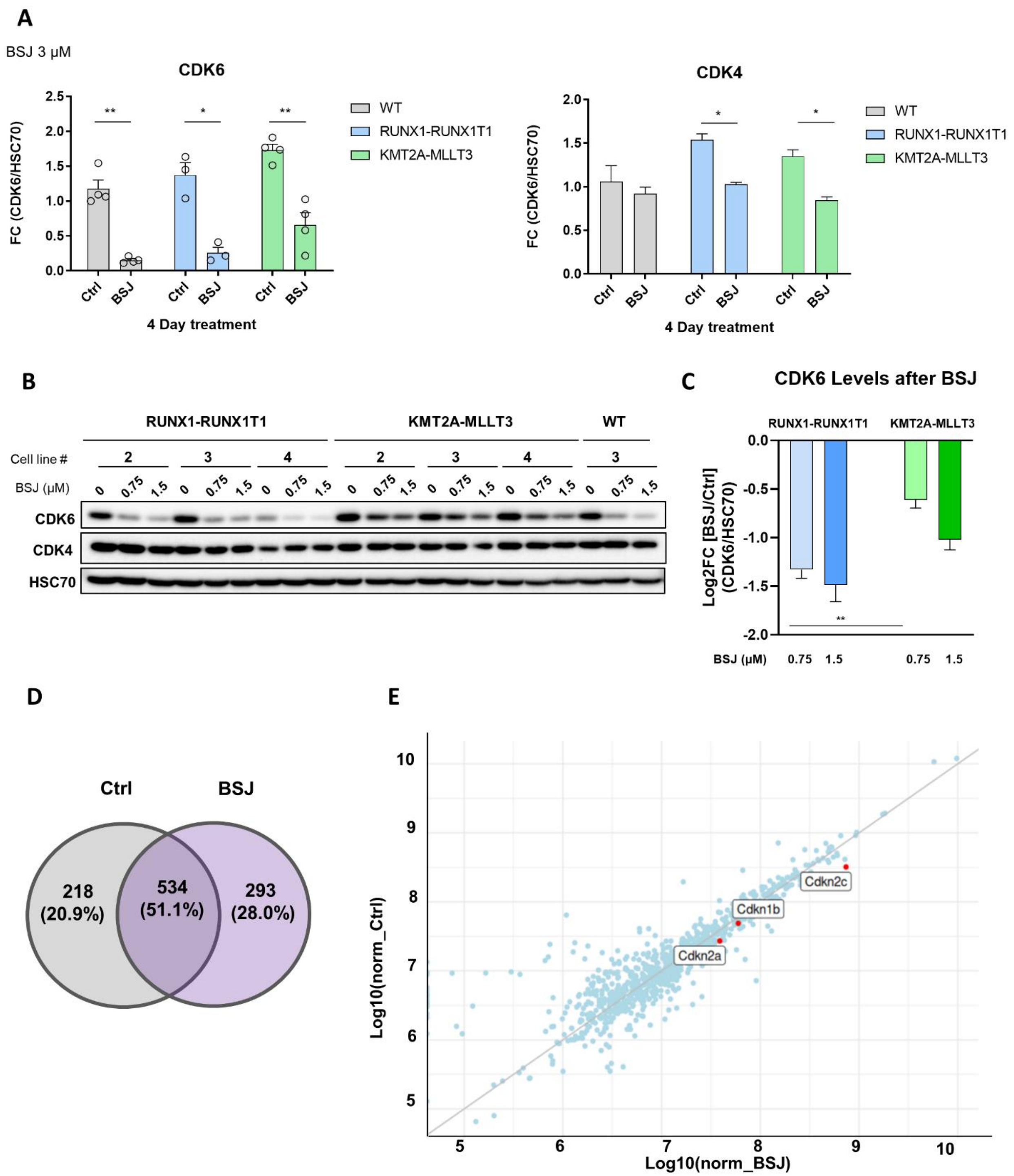

Supplementary Materials

F

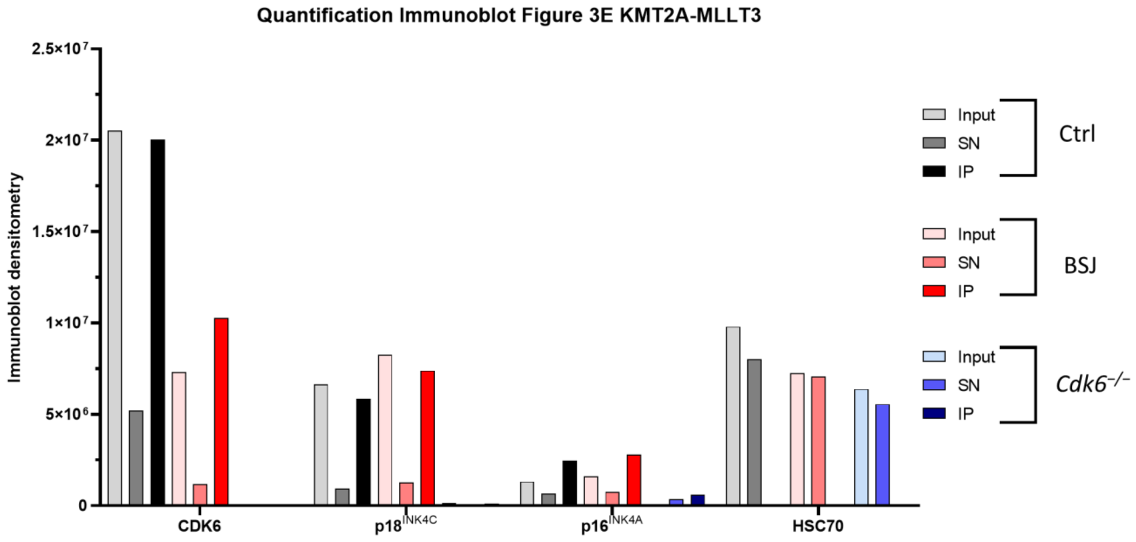

G

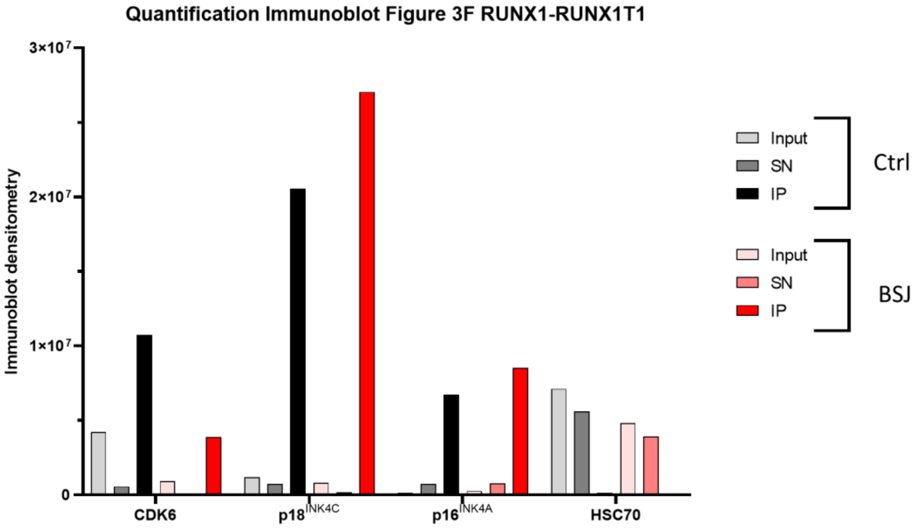

Figure S3. BSJ efficacy depends on INK4 protein levels. **(A)** Immunoblot quantification of CDK6 (left) and CDK4 (right) normalized to HSC70 from HPC^{LSK} WT (n=4), RUNX1-RUNX1T1 (n=3) and KMT2A-MLLT3 (n=4) cells treated with 3 µM BSJ or vehicle control for 4 days (left). **p* <0.05, ***p* <0.01. **(B)** Immunoblot for CDK6, CDK4 and HSC70 as loading control of RUNX1-RUNX1T1+ (n=3), KMT2A-MLLT3+ (n=3) and WT (n=1) cells treated with 0.75 µM BSJ, 1.5 µM BSJ or vehicle ctrl for 24 h. **(C)** Log2 fold change (FC) (BSJ/Ctrl) of immunoblot quantification from (B) was calculated. ***p* = 0.0045. **(D)** Venn diagram of Mass Spectrometry experiment. Enriched proteins over the *Cdk6*$^{-/-}$ background control were calculated and percentage of proteins in the Ctrl sample, in the BSJ samples and both are shown. **(E)** Scatter plot of enriched proteins over the negative ctrl (*Cdk6*$^{-/-}$ =0). X-axis represents Log10 normalized abundances of BSJ sample and y-axis Log10 normalized abundances of Ctrl sample. **(F)** Immunoblot densitometry quantification of Figure 3E. **(G)** Immunoblot densitometry quantification of Figure 3F.

Supplementary Materials

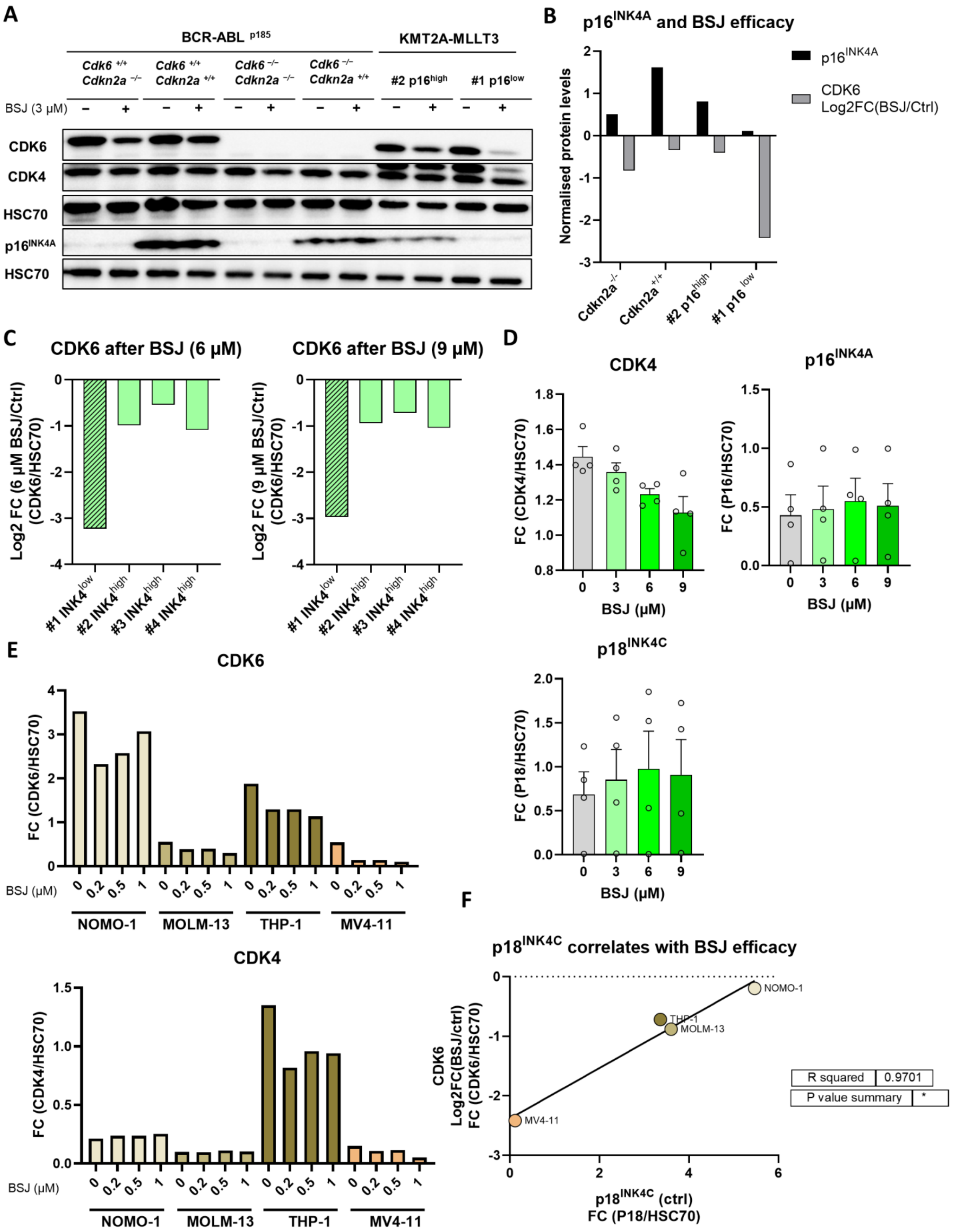

Supplementary Materials

Figure S4. INK4 levels as predictive markers for CDK6 degrader. **(A)** Immunoblot for CDK6, CDK4, p16^{INK4A} and HSC70 of *Cdkn2a*$^{-/-}$ and *Cdk6*$^{-/-}$ BCR-ABL^{p185+} cells with and without re-expression of *Cdkn2a* and *Cdk6* and KMT2A-MLLT3+ INK4^{high} and INK4^{low} cells after treatment with 3 μM BSJ or vehicle control for 24 h. **(B)** Immunoblot quantification from (A) of BCR-ABL^{p185+} *Cdkn2a*$^{+/+}$ and *Cdkn2a*$^{-/-}$ cells and KMT2A-MLLT3+ INK4^{high} and INK4^{low} cells. P16^{INK4A} levels of the vehicle treated samples relative to HSC70 compared to the Log2 FC (BSJ/Ctrl) of CDK6 levels. **(C)** Log2FC (BSJ/Ctrl) from immunoblot quantification of CDK6 normalized to HSC70 of KMT2A-MLLT3 samples grouped into INK4^{low} and INK4^{high}. **(D)** Immunoblot quantification for CDK4 (top left panel) p16^{INK4A} (top right panel) and p18^{INK4C} (bottom panel), normalized to HSC70 of KMT2A-MLLT3+ cells (n=4) treated with increasing concentrations (3 μM, 6 μM and 9 μM) of BSJ or vehicle control for four days. **(E)** Immunoblot quantification of the human AML cell lines positive for KMT2A-MLLT3 (NOMO-1, MOLM-13 and THP-1) or KMT2A-AFF1 (MV4-11) treated with increasing concentrations of BSJ (0.2 μM, 0.5 μM and 1 μM) or vehicle control for 90 minutes. CDK6 levels (top panel) and CDK4 levels (bottom panel) normalized to HSC70 are shown. **(F)** Correlation of p18^{INK4C} levels and CDK6 Log2 FC (BSJ/Ctrl) from immunoblot quantification is shown. R^2=0.9701; *p = 0.0151.

Original
uncropped immu

Figure S5. Original uncropped immunoblot scans

Supplementary Materials

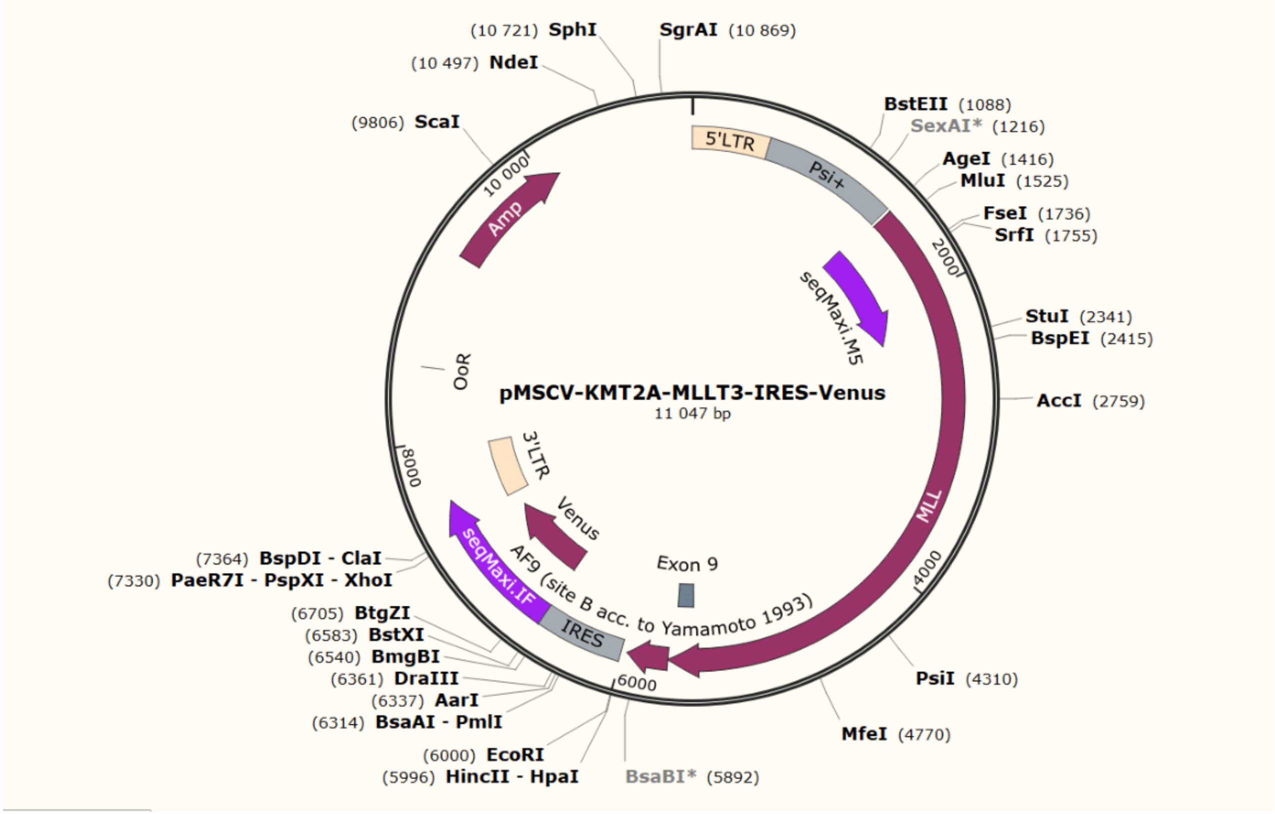

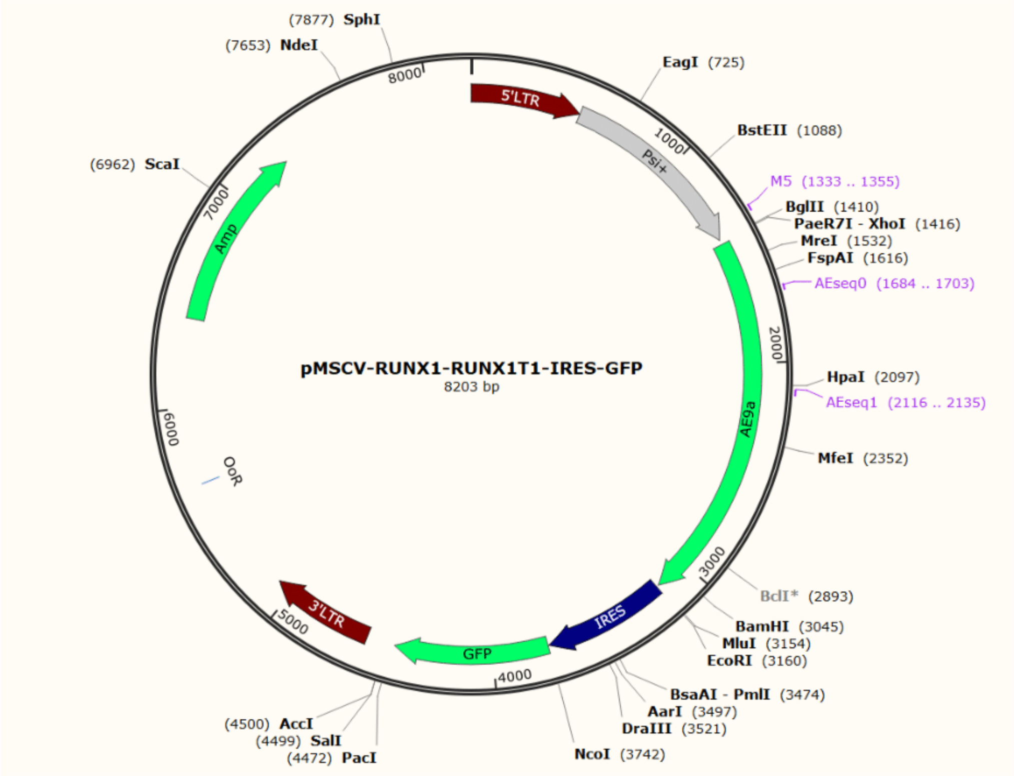

Figure S6. Plasmid maps

Supplementary Materials

Supplementary Methods

Table S1. Primer sequences for qPCRs

Target gene	Primer	Sequence (5′-3′)
Rplp0 (murine)	Forward	GCTTTCTGGAGGGTGTCC
	Reverse	GCTTCAGCTTTGGCAGGG
RUNX1-RUNX1T1 (human)	Forward	CACCTACCACAGAGCCATCAAA
	Reverse	ATCCACAGGTGAGTCTGGCATT
KMT2A-MLLT3 (human)	Forward	TGTGAAGCAGAAATGTGTGG
	Reverse	TGCCTTGTCACATTCACCAT

Table S2. Antibodies used in this study:

Target	Description/Company	Dilution/concentration
CDK6 (Westernblot)	DCS-90 SC-56282 Santa Cruz	1:1000
CDK6 (IP)	C-21 SC-177 Santa Cruz	1μg Antibody / 37.5 μg protein
CDK4	C-22 SC-260 Santa Cruz	1:1000
P16 ^{INK4A} (Cdkn2a)	Ab211542 Abcam	1:2000
P18 ^{INK4C} (Cdkn2c)	EPR15891 Ab192239 Abcam	1:1000
HSC70	SC-7298 Santa Cruz	1:2000
Anti-mouse IgG HRP-linked	7076S Cell Signaling	1:5000
Anti-rabbit IgG HRP-linked	7074S Cell Signaling	1:5000
Gr1 (FACS) : Brilliant Violet 650 anti-mouse Ly-6G Rat IgG2a, kappa	Clone: 1A8 BioLegend	1:100

Liquid chromatography coupled to tandem mass spectrometry (LC-MS/MS)

Eluates were processed using an adapted Single-Pot solid-phase-enhanced sample preparation (SP3) methodology [1]. Briefly, equal volumes (125 µl containing 6250 µg) of two different kind of paramagnetic carboxylate modified particles (SpeedBeads 45152105050250 and 65152105050250; GE Healthcare) were mixed, washed three times with 250 µl water and reconstituted to a final concentration of 50 µg/µl with LC-MS grade water (LiChrosolv; MERCK KgaA). Samples (50 µL) were filled up to 100 µL with 2% SDS, 100 mM HEPES, pH 8.0, and proteins were reduced with a final concentration of 10 mM DTT and incubated at 56°C for 1 hour. After cooling down to room temperature, reduced cysteines were alkylated with iodoacetamide at a final concentration of 55 mM for 30 min in the dark. For tryptic digestion, 400 µg of mixed beads were added to reduced and alkylated samples, vortexed gently and incubated for 5 minutes at room temperature. The formed particles-protein complexes were precipitated by addition of acetonitrile to a final concentration of 70% [V/V], mixed briefly via

pipetting before incubating for 18 minutes at room temperature. Particles were then immobilized using a magnetic rack (DynaMag-2 Magnet; Thermo Fisher Scientific) and supernatant was discarded. SDS was removed by washing two times with 200 µl 70% ethanol and one time with 180 µl 100MS% acetonitrile. After removal of organic solvent, particles were resuspended in 100 µl of 50 mM NH4HCO3 and samples digested by incubating with 2 µg of Trypsin overnight at 37°C. Samples were acidified to a final concentration of 1% Trifluoroacetic acid (Uvasol; MERCK KgaA) prior to immobilizing the beads on the magnetic rack. Samples were then desalted and concentrated using stage tips with two stacked C18 plugs (Empore; MERCK KgaA) [2]. Stage tips were activated with three times 100 µl acetonitrile and equilibrated with three times 100 µl of 0.4% formic acid, 2% TFA in water before loading the samples. Salts were cleaned up with 100 µl of 0.1% TFA and peptides were eluted using two times 50 µl 90% acetonitrile, 0.4% formic acid. Finally, eluates were dried in a vacuum concentrator and reconstituted in 10 µl of 0.1% TFA.

Mass spectrometry was performed on an Orbitrap Fusion Lumos mass spectrometer (ThermoFisher Scientific, San Jose, CA) coupled to an Dionex Ultimate 3000RSLC nano system (ThermoFisher Scientific, San Jose, CA) via nanoflex source interface. Tryptic peptides were loaded onto a trap column (Pepmap 100 5 µm, 5 × 0.3 mm, ThermoFisher Scientific, San Jose, CA) at a flow rate of 10 µL/min using 0.1% TFA as loading buffer. After loading, the trap column was switched in-line with a 50 cm, 75 µm inner diameter analytical column (packed in-house with ReproSil-Pur 120 C18-AQ, 3 µm, Dr. Maisch, Ammerbuch-Entringen, Germany). Mobile-phase A consisted of 0.4% formic acid in water and mobile-phase B of 0.4% formic acid in a mix of 90% acetonitrile and 10% water. The flow rate was set to 230 nL/min and a 90 min gradient used (4 to 24% solvent B within 82 min, 24 to 36% solvent B within 8 min and, 36 to 100% solvent B within 1 min, 100% solvent B for 6 min before bringing back solvent B at 4% within 1 min and equilibrating for 18 min). Analysis was performed in a data-dependent acquisition mode. Full MS scans were acquired with a mass range of 375 - 1650 m/z in the orbitrap at a resolution of 120,000 (at 200 m/z). Monoisotopic peak determination was set to peptides with inclusion of charge states between 2 and 5, Automatic gain control (AGC) was set to a target of 2×10^5, and a maximum injection time of 50 ms was applied. Precursor ions for MS2 analysis were selected using a TopN dependent scan approach with a cycle time of up to 20 scans. MS2 spectra were acquired in the orbitrap (FT) using a quadrupole isolation window of 1.6 Da and higher energy collision induced dissociation (HCD) at a fixed normalized collision energy (NCE) of 30%. The resolution was set to 15,000 (at 200 m/z) with a fixed first mass of 120 m/z, an AGC target set to 5×10^4, and a maximum injection time set to 100 ms. Dynamic exclusion for selected ions was 30 s, and isotopes were excluded. An unscheduled inclusion mass list (550 m/z values) was built using Skyline version 20.2.0.343 for 19 proteins of interest (P51480, Q64364, P39689, P46414, P30280, P30282, P30285, P54071, Q8CIG8, Q4JK59, P25322, Q61937, P02340, P42227, P05627, O89090, P13864, O88508, O88509). The option 'Perform

dependent scan on most intense ion if no target ions are found' was enabled. A single lock mass at m/z 445.120024 [3] was employed. Xcalibur version 4.3.73.11 and Tune 3.4.3072.18 were used to operate the instrument.

Acquired raw data files were processed using the Proteome Discoverer 2.4.1.15 platform, utilizing the database search engine Sequest HT. Percolator V3.0 was used to remove false positives with a false discovery rate (FDR) of 1% on PSM, peptide and protein level under strict conditions. Searches were performed with full tryptic digestion against the mouse SwissProt database v2020.01 (25,097 sequences plus 298 appended known contaminants) with up to two miscleavage sites. Oxidation (+15.9949 Da) of methionine, phosphorylation (+79.9663 Da) of serine, threonine or tyrosine was set as variable modification, whilst carbamidomethylation (+57.0214 Da) of cysteine residues was set as fixed modifications. For the protein N-terminus, acetylation of methionine (+42.0110 Da), and loss of methionine with or without acetylation (respectively -89.030 Da and -131.040 Da) was set as variable modification. Data was searched with mass tolerances of ±10 ppm on the precursor ions and 0.025 Da on the fragment ions. Results were filtered to include peptide spectrum matches (PSMs) with Sequest HT cross-correlation factor (Xcorr) scores of ≥1 and high peptide confidence, as well as ptmRS site probabilities of ≥75. Only proteins were included that were identified by at least two peptides and one unique peptide sequence. For calculation of protein areas, Minora Feature Detector node and Precursor Ions Quantifier node, both integrated in Thermo Proteome Discoverer were used. Automated chromatographic peak alignment and feature linking mapping were enabled with total peptide amount used for normalization between individual runs. Protein abundances were calculated using sum intensities of respective peptide features including only unique peptide groups. Protein ratios were generated using the non-nested approach and p-value calculation was performed applying t-test and Benjamini-Hochberg correction. Normalized abundances and log2 transformed fold-changes (log2FC) for Ctrl/$Cdk6^{-/-}$ or BSJ/$Cdk6^{-/-}$ were calculated and plotted. The mass spectrometry data has been deposited to the ProteomXchange Consortium via the PRIDE partner repository [4] with the dataset identifier PXD-.

References

1. Hughes, C.S.; Foehr, S.; Garfield, D.A.; Furlong, E.E.; Steinmetz, L.M.; Krijgsveld, J. Ultrasensitive proteome analysis using paramagnetic bead technology. *Mol. Syst. Biol.* **2014**, *10*, 757, doi:10.15252/msb.20145625.
2. Rappsilber, J.; Mann, M.; Ishihama, Y. Protocol for micro-purification, enrichment, pre-fractionation and storage of peptides for proteomics using StageTips. *Nat. Protoc.* **2007**, *2*, 1896–1906, doi:10.1038/nprot.2007.261.
3. Olsen, J.V.; Godoy, L.M.F. de; Li, G.; Macek, B.; Mortensen, P.; Pesch, R.; Makarov, A.; Lange, O.; Horning, S.; Mann, M. Parts per million mass accuracy on an Orbitrap mass

spectrometer via lock mass injection into a C-trap. *Mol. Cell. Proteomics* **2005**, *4*, 2010–2021, doi:10.1074/mcp.T500030-MCP200.

4. Perez-Riverol, Y.; Csordas, A.; Bai, J.; Bernal-Llinares, M.; Hewapathirana, S.; Kundu, D.J.; Inuganti, A.; Griss, J.; Mayer, G.; Eisenacher, M.; et al. The PRIDE database and related tools and resources in 2019: improving support for quantification data. *Nucleic Acids Res.* **2019**, *47*, D442-D450, doi:10.1093/nar/gky1106.

Table S3: Raw and normalised mass spectrometry (LC-MS/MS) data.

Raw and
normalised mass
