# Supplementary material for: CDK6 Degradation Is Counteracted by p16INK4A and p18INK4C in AML
Source: Cancers (Basel). 2022 Mar 18;14(6):1554. doi: 10.3390/cancers14061554 (PMC8946512; doi:10.3390/cancers14061554)

Figure 1D

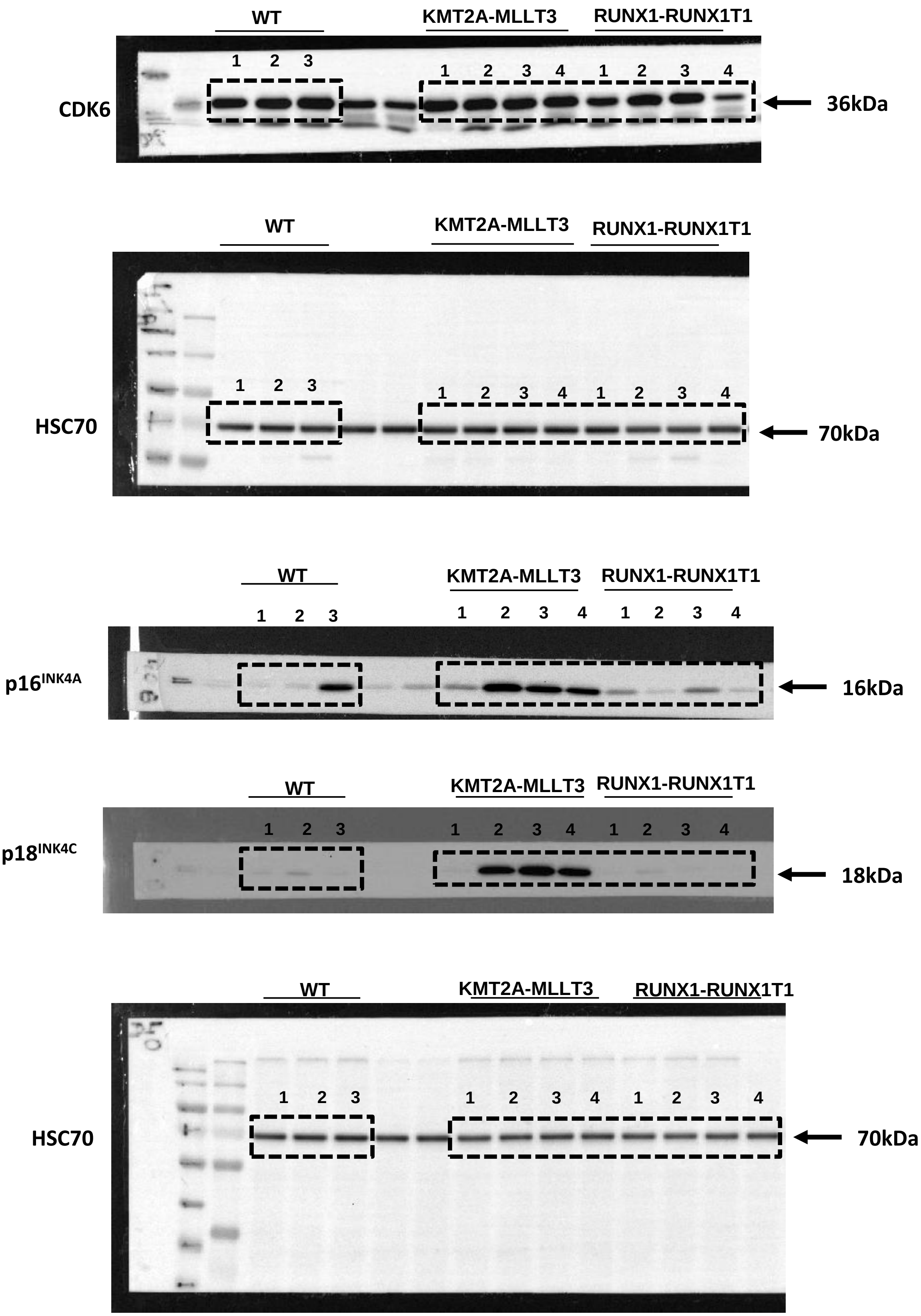

WT
KMT2A-MLLT3
RUNX1-RUNX1T1
1 2 3
1 2 3 4 1 2 3 4
CDK6
36kDa
WT
KMT2A-MLLT3
RUNX1-RUNX1T1
1 2 3
1 2 3 4 1 2 3 4
HSC70
70kDa
WT
KMT2A-MLLT3
RUNX1-RUNX1T1
1 2 3
1 2 3 4 1 2 3 4
p16INK4A
16kDa
WT
KMT2A-MLLT3
RUNX1-RUNX1T1
1 2 3
1 2 3 4 1 2 3 4
p18INK4C
18kDa
WT
KMT2A-MLLT3
RUNX1-RUNX1T1
1 2 3
1 2 3 4 1 2 3 4
HSC70
70kDa

Figure S2A

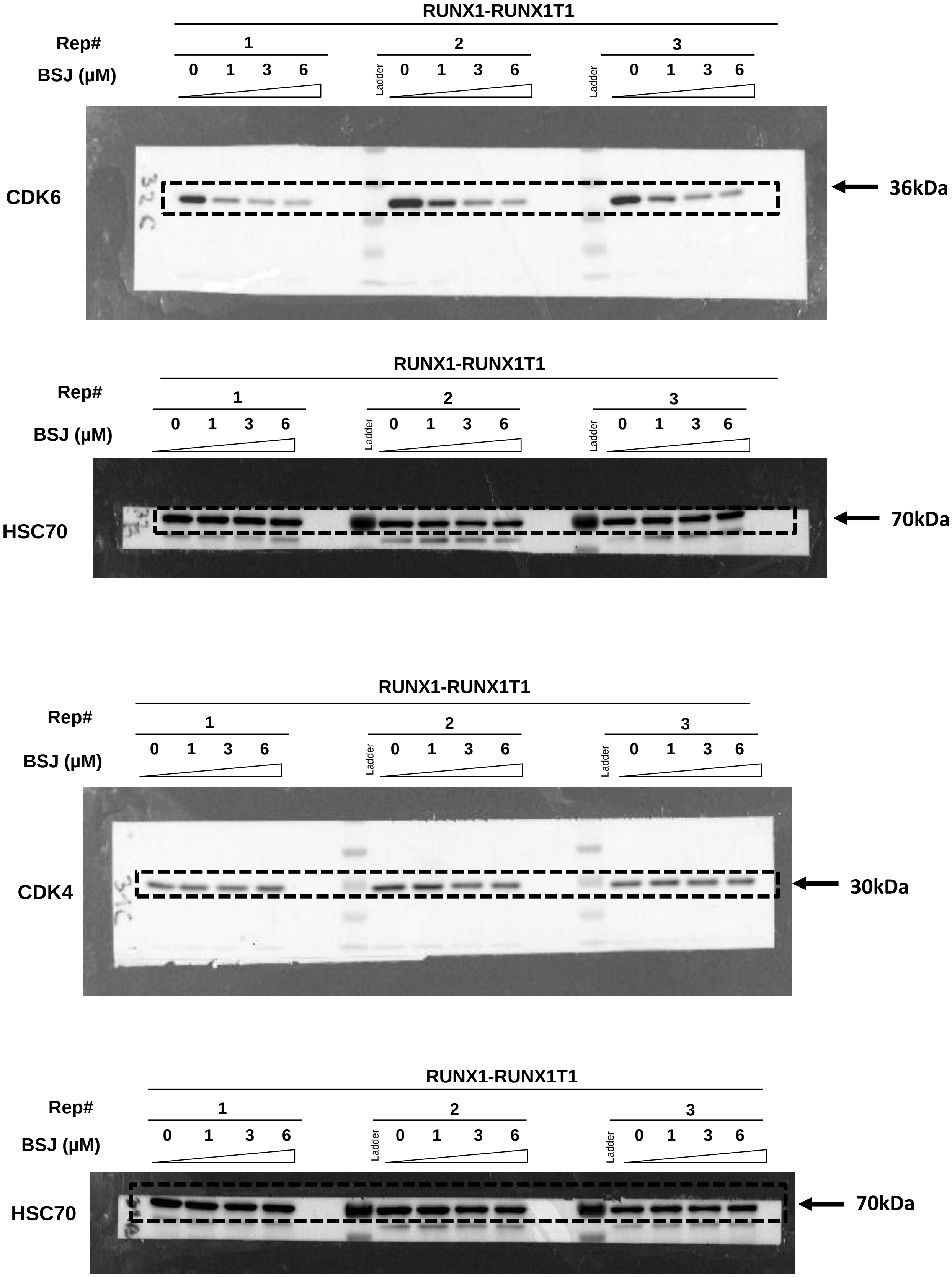
RUNX1-RUNX1T1
Rep#
1
2
3
BSJ (μM)
0 1 3 6
Ladder
0 1 3 6
Ladder
0 1 3 6
CDK6
36kDa
RUNX1-RUNX1T1
Rep#
1
2
3
BSJ (μM)
0 1 3 6
Ladder
0 1 3 6
Ladder
0 1 3 6
HSC70
70kDa
RUNX1-RUNX1T1
Rep#
1
2
3
BSJ (μM)
0 1 3 6
Ladder
0 1 3 6
Ladder
0 1 3 6
CDK4
30kDa
RUNX1-RUNX1T1
Rep#
1
2
3
BSJ (μM)
0 1 3 6
Ladder
0 1 3 6
Ladder
0 1 3 6
HSC70
70kDa

Figure S2C

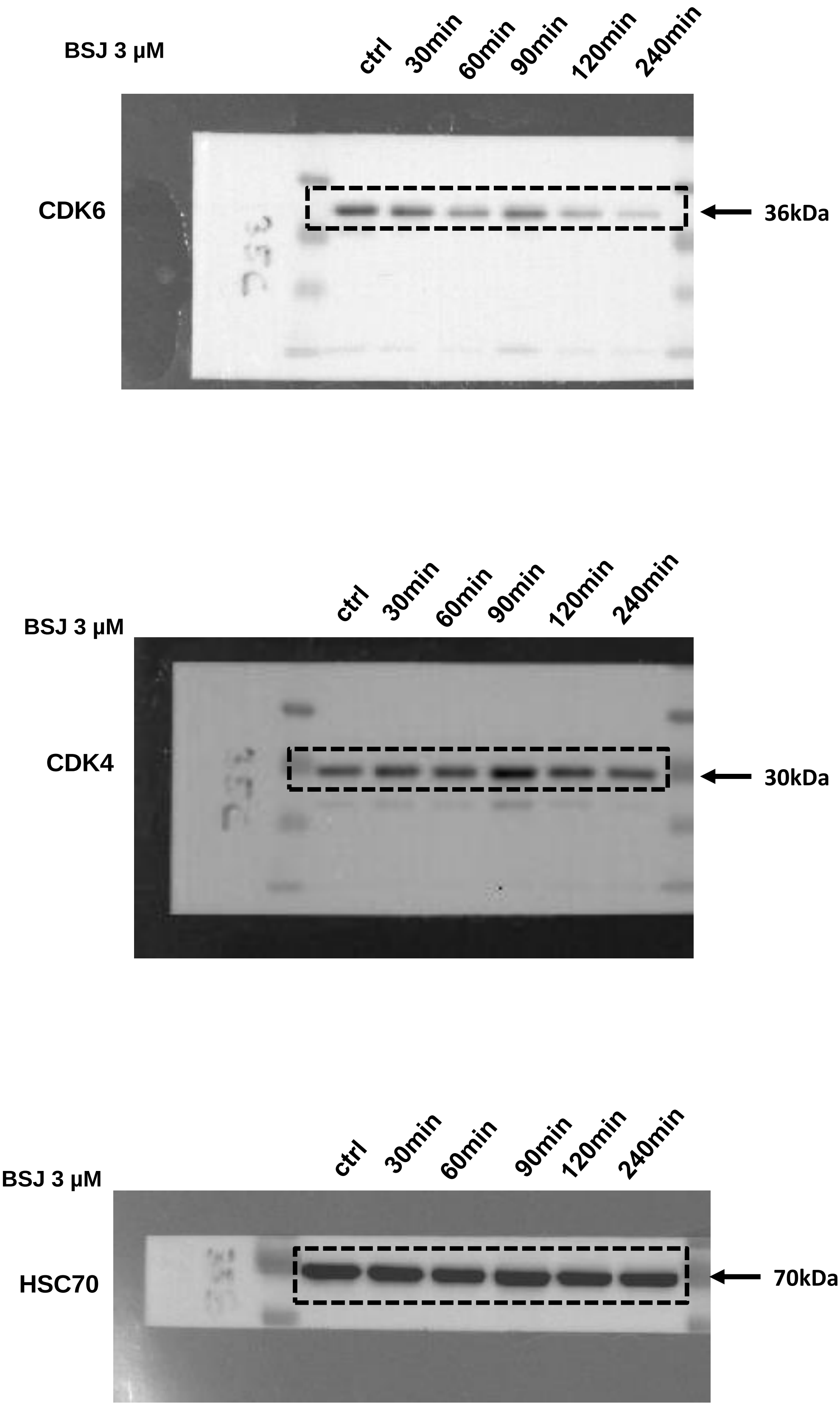
BSJ 3 µM
ctrl
30min
60min
90min
120min
240min
CDK6
36kDa
BSJ 3 µM
ctrl
30min
60min
90min
120min
240min
CDK4
30kDa
BSJ 3 µM
ctrl
30min
60min
90min
120min
240min
HSC70
70kDa

Figure 3A

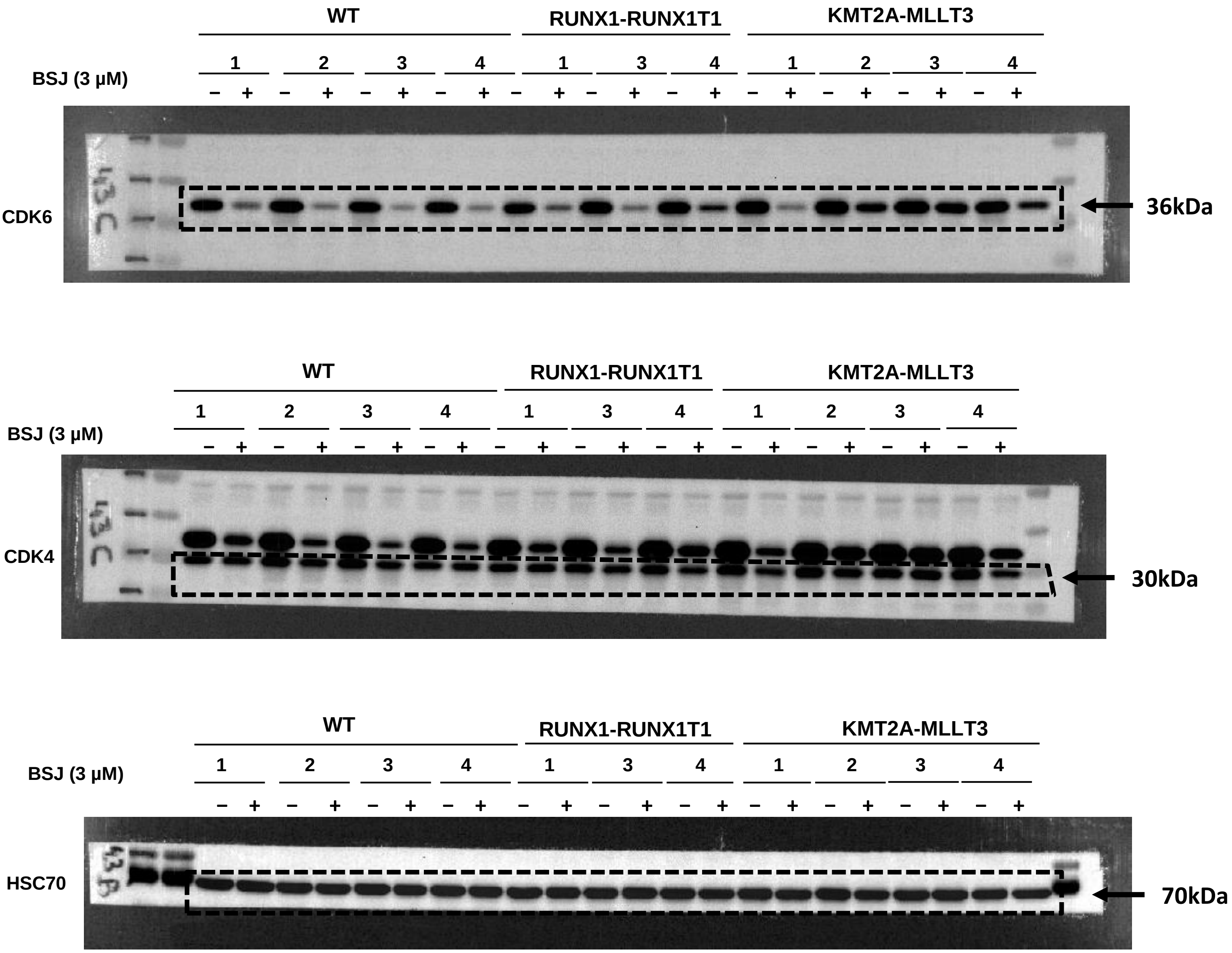

WT
RUNX1-RUNX1T1
KMT2A-MLLT3
1 2 3 4 1 3 4 1 2 3 4
BSJ (3 μM)
– + – + – + – + – + – + – + – + – + – + – +
CDK6
36kDa
WT
RUNX1-RUNX1T1
KMT2A-MLLT3
1 2 3 4 1 3 4 1 2 3 4
BSJ (3 μM)
– + – + – + – + – + – + – + – + – + – + – +
CDK4
30kDa
WT
RUNX1-RUNX1T1
KMT2A-MLLT3
1 2 3 4 1 3 4 1 2 3 4
BSJ (3 μM)
– + – + – + – + – + – + – + – + – + – + – +
HSC70
70kDa

Figure 3E

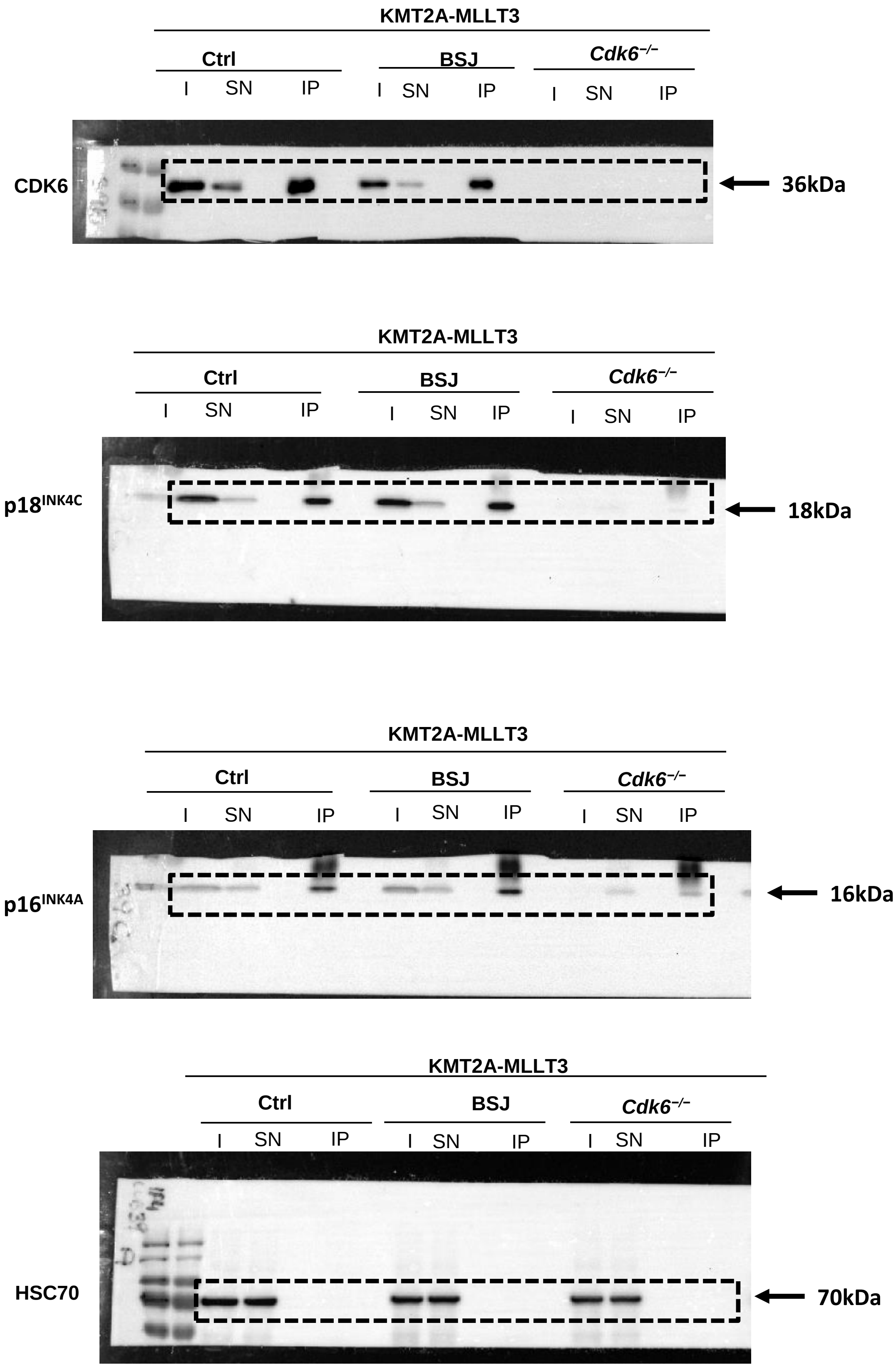
KMT2A-MLLT3
Ctrl
BSJ
Cdk6−/−
I SN IP I SN IP I SN IP
CDK6
36kDa
KMT2A-MLLT3
Ctrl
BSJ
Cdk6−/−
I SN IP I SN IP I SN IP
p18INK4C
18kDa
KMT2A-MLLT3
Ctrl
BSJ
Cdk6−/−
I SN IP I SN IP I SN IP
p16INK4A
16kDa
KMT2A-MLLT3
Ctrl
BSJ
Cdk6−/−
I SN IP I SN IP I SN IP
HSC70
70kDa

Figure 3F

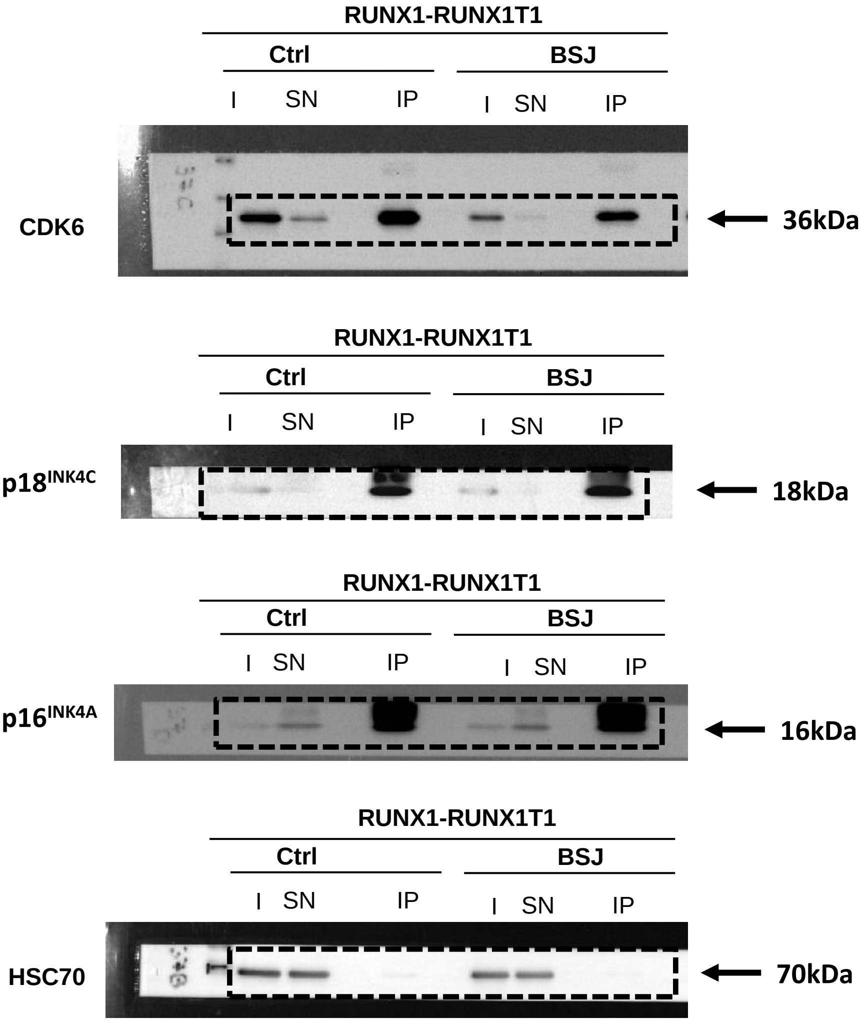

RUNX1-RUNX1T1
Ctrl
BSJ
I
SN
IP
I
SN
IP
CDK6
36kDa
RUNX1-RUNX1T1
Ctrl
BSJ
I
SN
IP
I
SN
IP
p18INK4C
18kDa
RUNX1-RUNX1T1
Ctrl
BSJ
I
SN
IP
I
SN
IP
p16INK4A
16kDa
RUNX1-RUNX1T1
Ctrl
BSJ
I
SN
IP
I
SN
IP
HSC70
70kDa

Figure S3B

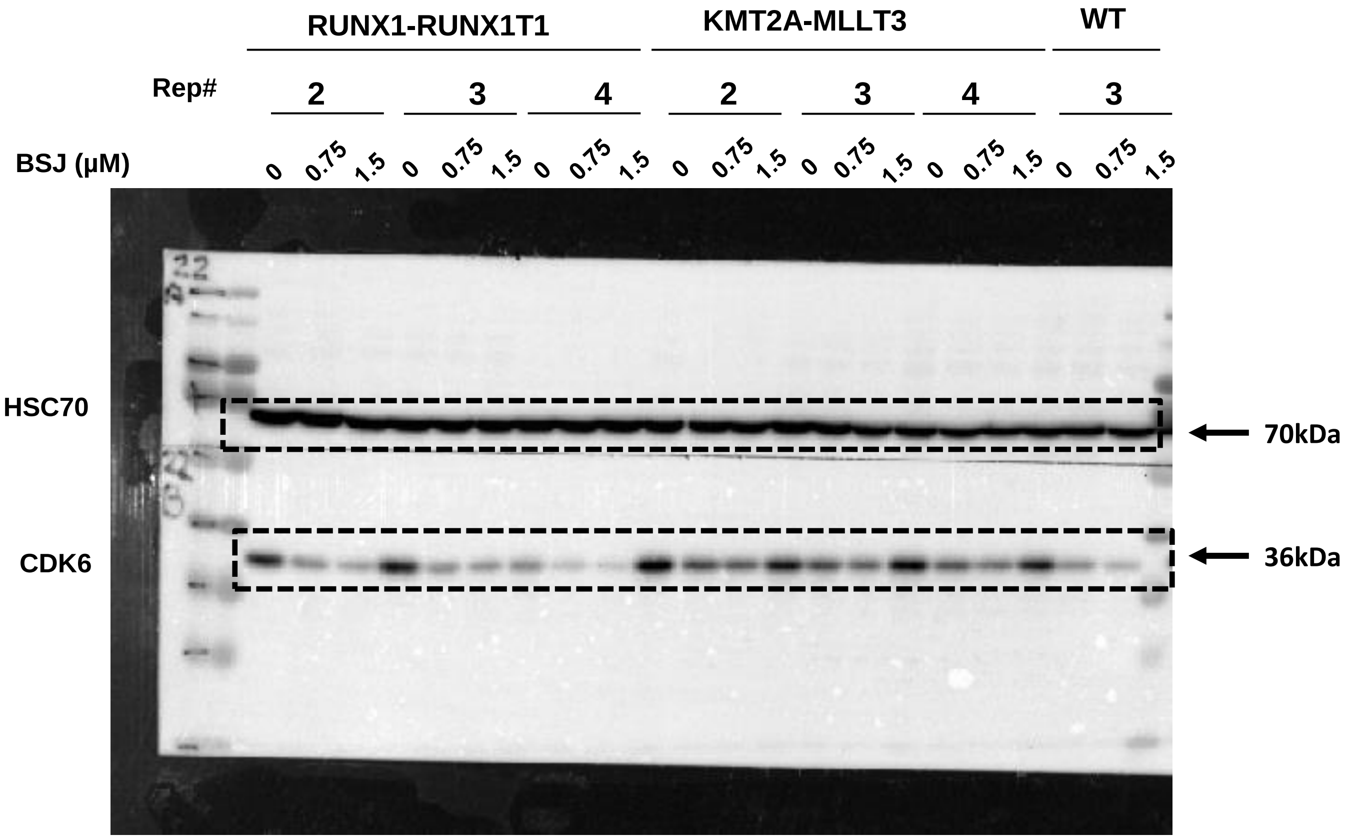
RUNX1-RUNX1T1
KMT2A-MLLT3
WT
Rep#
2
3
4
2
3
4
3
BSJ (μM)
0 0.75 1.5 0 0.75 1.5 0 0.75 1.5 0 0.75 1.5 0 0.75 1.5 0 0.75 1.5 0 0.75 1.5
HSC70
CDK6
70kDa
36kDa

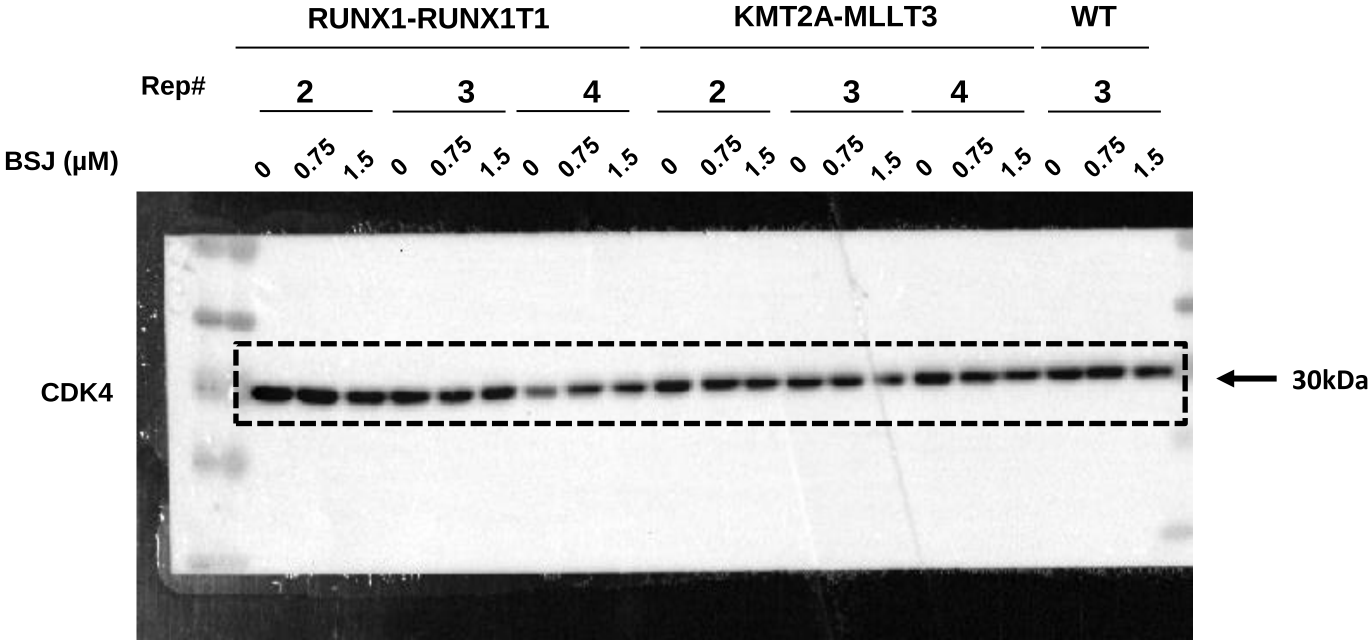
RUNX1-RUNX1T1
KMT2A-MLLT3
WT
Rep#
2
3
4
2
3
4
3
BSJ (μM)
0 0.75 1.5 0 0.75 1.5 0 0.75 1.5 0 0.75 1.5 0 0.75 1.5 0 0.75 1.5 0 0.75 1.5
CDK4
30kDa

Figure 4A

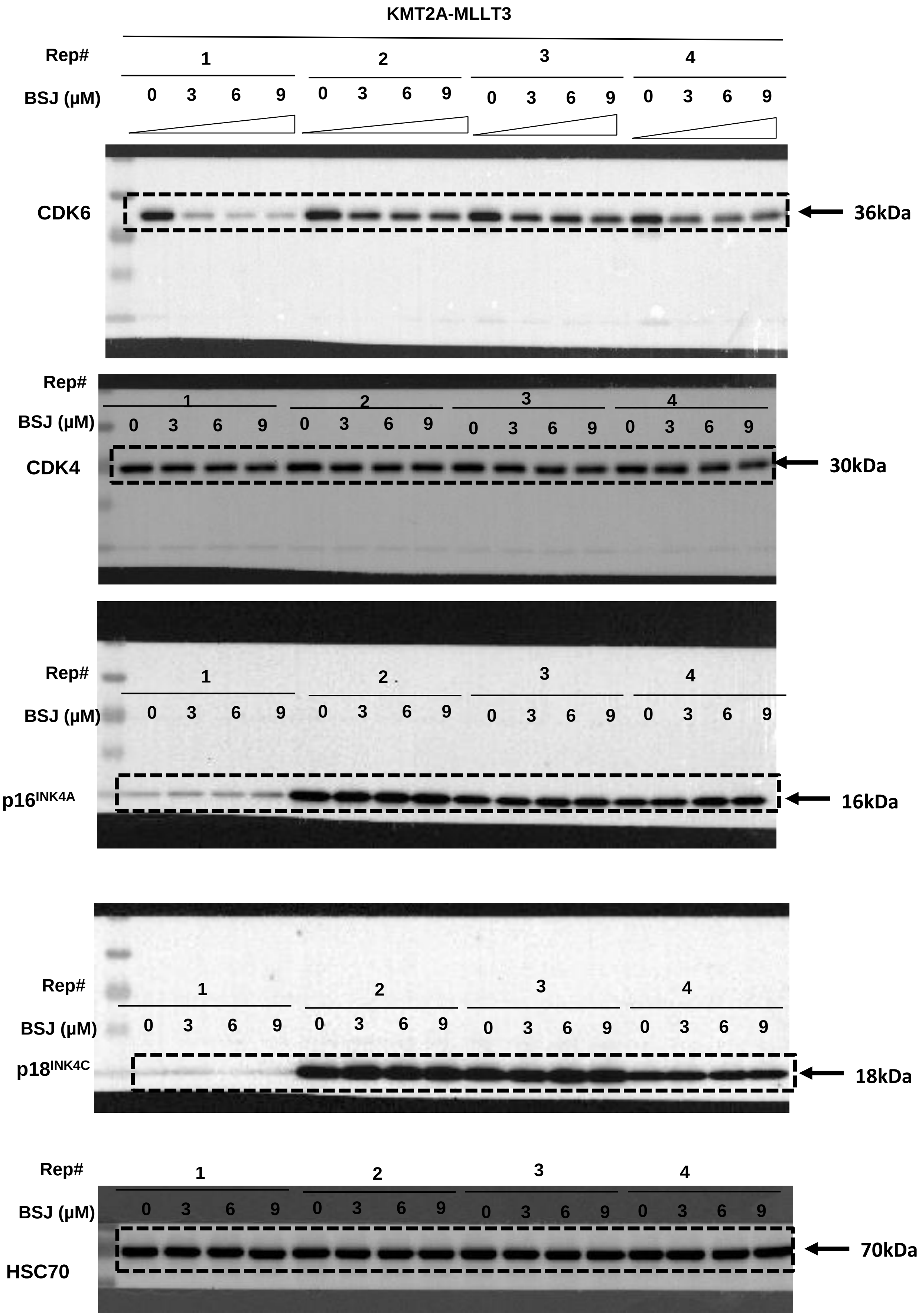
KMT2A-MLLT3
Rep#
1
2
3
4
BSJ (µM)
0 3 6 9 0 3 6 9 0 3 6 9 0 3 6 9
CDK6
36kDa
CDK4
30kDa
p16INK4A
16kDa
p18INK4C
18kDa
HSC70
70kDa

Figure 4D

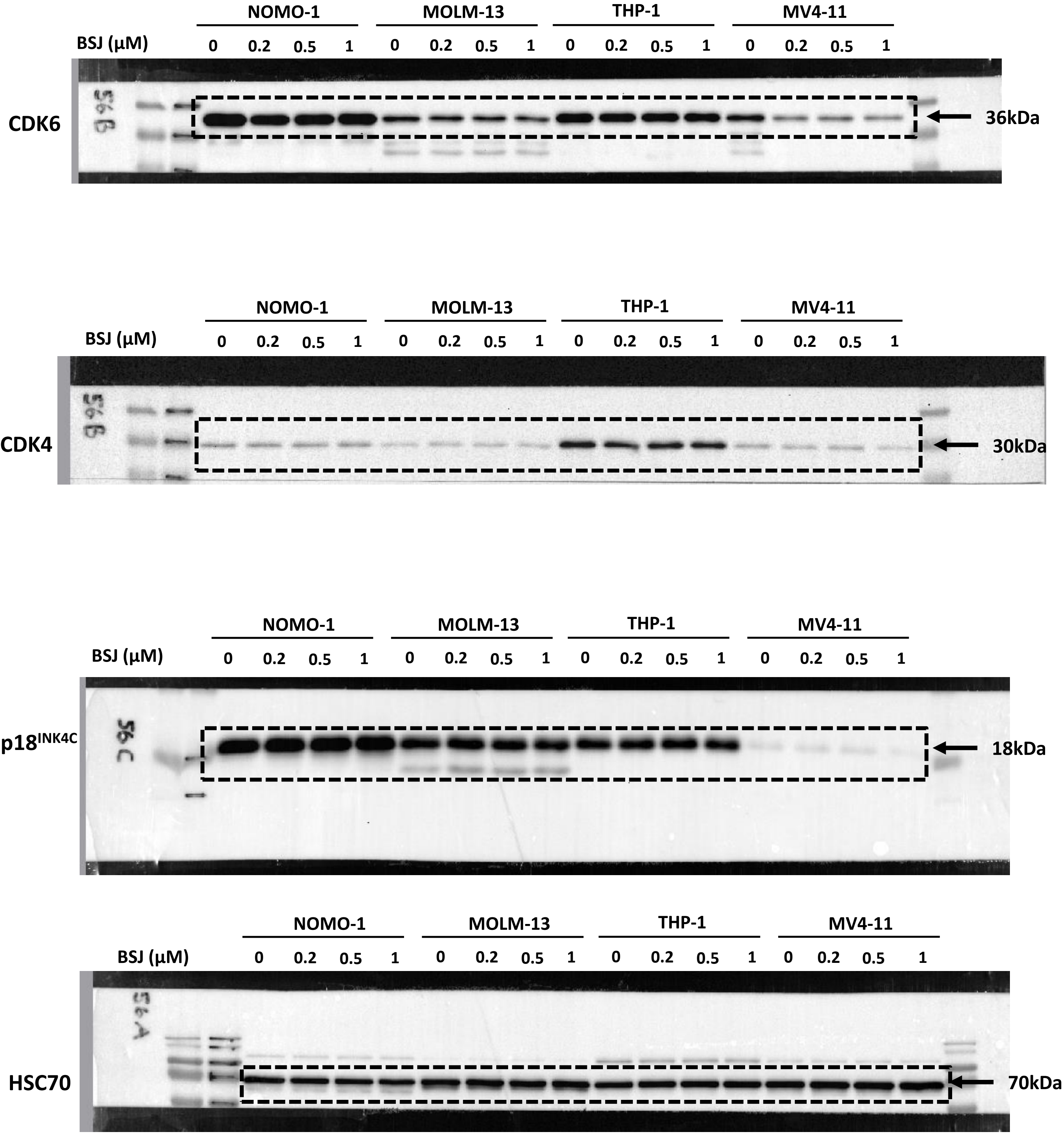

NOMO-1
MOLM-13
THP-1
MV4-11
BSJ (µM)
0 0.2 0.5 1 0 0.2 0.5 1 0 0.2 0.5 1 0 0.2 0.5 1
CDK6
36kDa
CDK4
30kDa
p18INK4C
18kDa
HSC70
70kDa

Figure S4A I

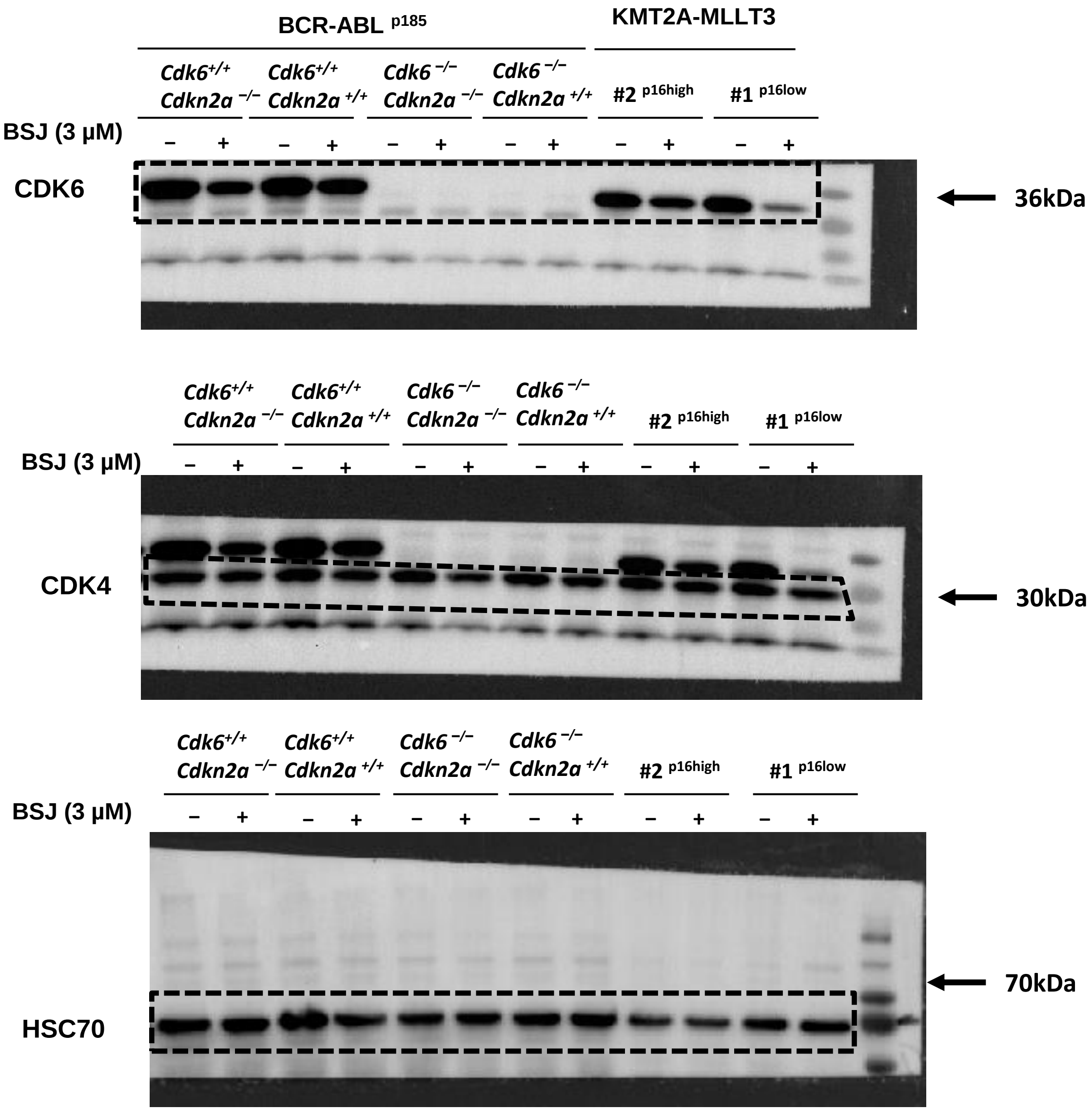

BCR-ABL p185
KMT2A-MLLT3
Cdk6+/+ Cdkn2a −/−
Cdk6+/+ Cdkn2a +/+
Cdk6 −/− Cdkn2a −/−
Cdk6 −/− Cdkn2a +/+
#2 p16high
#1 p16low
BSJ (3 µM)
− + − + − + − + − + − +
CDK6
36kDa
CDK4
30kDa
HSC70
70kDa

Figure S4A II

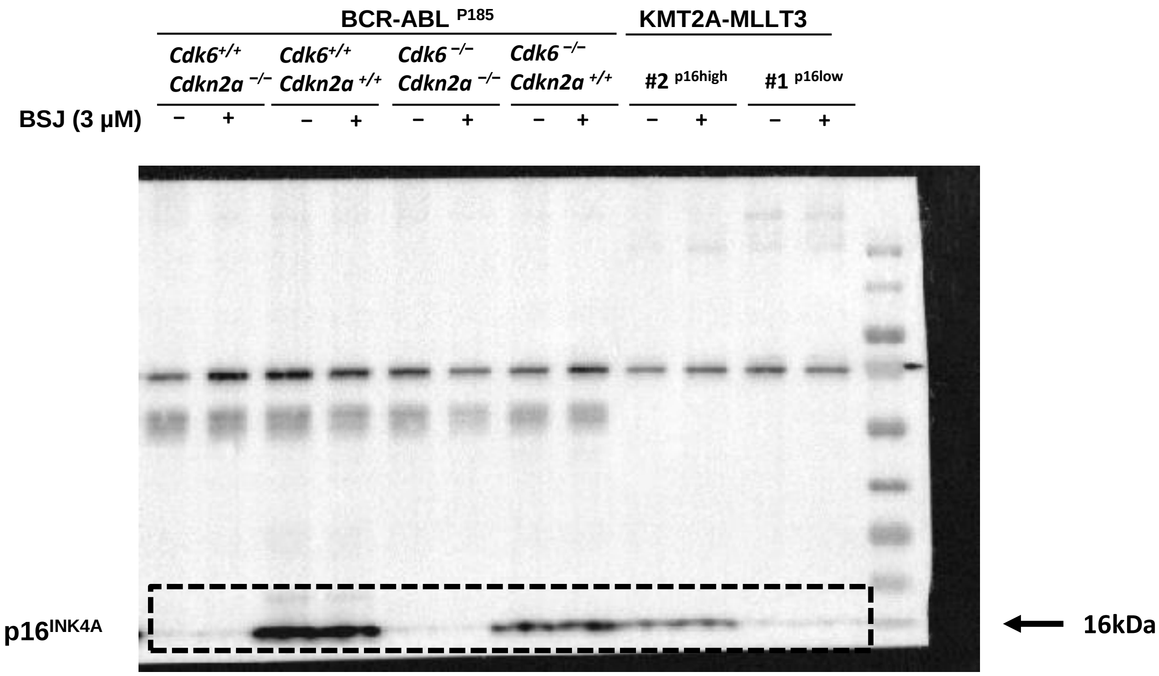

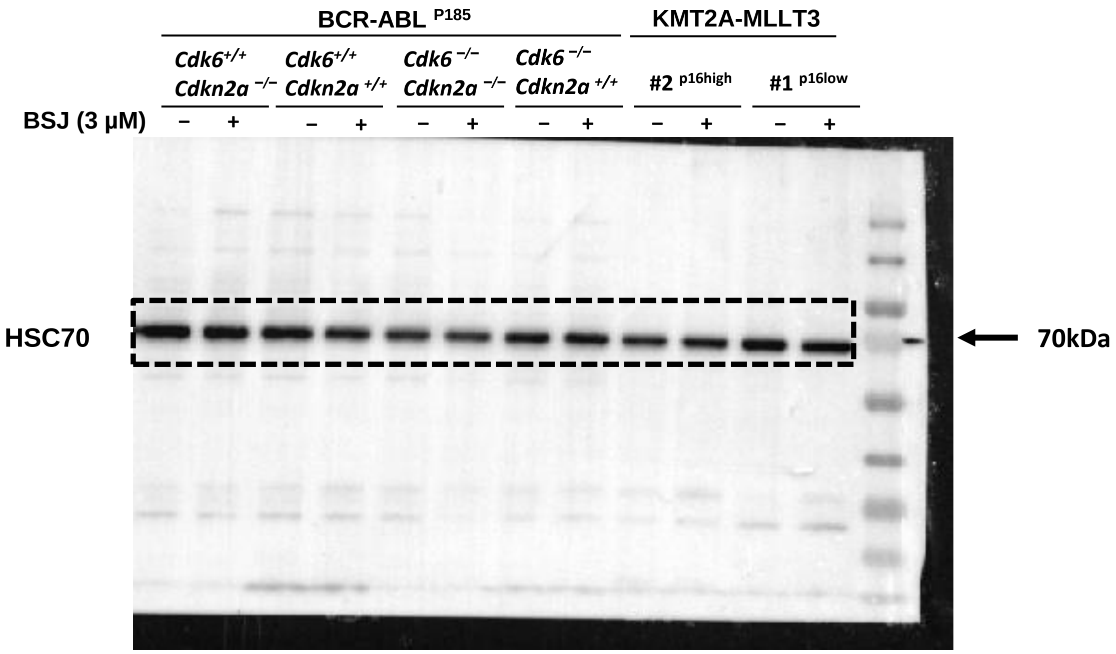

Supplement: Supplementary file 1 [file cancers-14-01554-s001.zip › Original uncropped immunoblot scans.pdf]
